# The Odd “RB” Phage—Identification of Arabinosylation as a New Epigenetic Modification of DNA in T4-Like Phage RB69

**DOI:** 10.3390/v10060313

**Published:** 2018-06-08

**Authors:** Julie A. Thomas, Jared Orwenyo, Lai-Xi Wang, Lindsay W. Black

**Affiliations:** 1 Department of Biochemistry and Molecular Biology, University of Maryland School of Medicine, 108 N. Greene St., Baltimore, MD 21201, USA; 2 Gosnell School of Life Sciences, Rochester Institute of Technology, 85 Lomb Memorial Drive, Rochester, NY 14623, USA; 3 Institute of Human Virology, University of Maryland School of Medicine, 725 West Lombard Street, Baltimore, MD 21201, USA; nyabutoo@gmail.com (J.O.); wang518@umd.edu (L.-X.W.); 4 Department of Chemistry and Biochemistry, University of Maryland, 8051 Regents Drive, College Park, MD 20742, USA

**Keywords:** epigenetic modification, restriction, T4 phage, RB69, hydroxymethylcytosine (hmC), glucosyl-hmC (ghmC), arabinosyl-hmC (ara-hmC), glucosyltransferase transferase, anion exchange chromatography, matrix assisted laser desorption ionization-time of flight (MALDI-TOF) mass spectrometry

## Abstract

In bacteriophages related to T4, hydroxymethylcytosine (hmC) is incorporated into the genomic DNA during DNA replication and is then further modified to glucosyl-hmC by phage-encoded glucosyltransferases. Previous studies have shown that RB69 shares a core set of genes with T4 and relatives. However, unlike the other “RB” phages, RB69 is unable to recombine its DNA with T4 or with the other “RB” isolates. In addition, despite having homologs to the T4 enzymes used to synthesize hmC, RB69 has no identified homolog to known glucosyltransferase genes. In this study we sought to understand the basis for RB69’s behavior using high-pH anion exchange chromatography (HPAEC) and mass spectrometry. Our analyses identified a novel phage epigenetic DNA sugar modification in RB69 DNA, which we have designated arabinosyl-hmC (ara-hmC). We sought a putative glucosyltranserase responsible for this novel modification and determined that RB69 also has a novel transferase gene, ORF003c, that is likely responsible for the arabinosyl-specific modification. We propose that ara-hmC was responsible for RB69 being unable to participate in genetic exchange with other hmC-containing T-even phages, and for its described incipient speciation. The RB69 ara-hmC also likely protects its DNA from some anti-phage type-IV restriction endonucleases. Several T4-related phages, such as *E. coli* phage JS09 and *Shigella* phage Shf125875 have homologs to RB69 ORF003c, suggesting the ara-hmC modification may be relatively common in T4-related phages, highlighting the importance of further work to understand the role of this modification and the biochemical pathway responsible for its production.

## 1. Introduction

T-even bacteriophages were the source of the first discovered epigenetic modifications of DNA—a significant finding as it formed the basis for the discovery of the first phage DNA-targeted host restriction–modification enzymes [1]. In T-even phages, all cytosine (C) positions in the genomic DNA are completely substituted by hydroxymethylcytosine (hmC) [2], which can be further modified, or “hypermodified”, by conversion to glucosyl-hmC (g-hmC). These g-hmC modifications are believed to function primarily to protect the phage DNA from restriction endonuclease (RE) attack, as evidenced by the number of enzymes unable to cleave T-even phage DNA [3,4]. In fact, g-hmC modification proved a major hindrance to initial efforts to sequence the T4 genome until the creation of T4 strains with multiple gene mutations for which DNAs have no modified cytosine (T4C) [3]. However, the g-hmC modification does not ensure escape from all restriction enzymes and recent studies have identified a remarkable diversity of type IV restriction enzymes that specifically target hmC and g-hmC modifications [5]. Illustrating the complexity of phage–bacterial interactions is the observation that specific protein inhibitors of type-IV REs that attack glucosylated hmC DNAs are injected by T-even phages into the *E. coli* cell with the genomic DNA [6,7]. Recent studies have also highlighted another likely advantage of g-hmC for evasion of host CRISPR-Cas systems [8,9].

The creation of T-even phage g-hmC is a multi-step process and differs from other well-known phage DNA modifications which result from a multitude of phage and host biochemical pathways [10]. T-even hmC is synthesized by a phage-encoded thymidylate synthase homolog (dCMP hydroxymethylase, gp42 [11,12]) which adds a hydroxymethyl group to deoxycytidine monophosphate. This is then incorporated into the genomic DNA during replication by the phage DNA polymerase gp43 [10]. The hmC is further modified to alpha- and beta- stereospecific glucosyl-hmC (g-hmC) by phage-encoded glucosyltransferases that employ uridine diphosphate glucose (UDP-glucose) synthesized by the *E. coli* host [13,14,15].

Among the “classical” T4, T2, and T6 phages that have been intensively studied at the enzyme and biochemical level, it has been reported that the hmC glucose modifications vary in extent (T4, 100%, T2 and T6, 75% hmC modification) as well as type of linkages [16]. T4 DNA has 70% alpha- and 30% beta-stereospecific glucosyl hmC produced by its alpha-glucosyltransferase (a-gt) and beta-glucosyltransferase (b-gt), respectively. The T4 hmC is first modified by its a-gt DNA immediately after replication (as inferred by its interactions with gp45, the replicative sliding clamp) but as the a-gt is unable to modify neighboring hmC residues, these unmodified residues are then modified by the b-gt [17]. T2 and T6 have β-1,6-glucosyl-α-glucose (gentiobiosyl) hmC (72% in T6 and 5% in T2) produced by homologous beta-alpha glucosyltransferases (ba-gt), as reviewed in [18]. Despite these g-hmC variations between T4, T2, and T6, viable genetic crosses can easily be made between these three phages.

Based on the characterization of T2, T4, and T6, the DNA modifications in other closely related bacteriophages with hmC DNAs have generally been supposed to also be glucosylations, although their chemistries have only been poorly if at all characterized. However, reflecting on the diversity of DNA modifications that have been identified in the small sample size of T4-related phages for which DNAs have been studied, we hypothesized that there may be a greater diversity of sugar modifications in these phages than previously realized. We were particularly intrigued by the *E. coli* phage RB69—one of the famous “RB” phages isolated by Rosina Berry from sewage from Long Island, NY, USA [19]—which is presumed to have modified DNA despite having no reported glucosyltransferases [10]. That RB69 DNA is modified has been inferred from it having homologs to T4’s two hydroxymethyltransferases (dCMP-hydroxymethylase and dCTPase-dUTPase) [10], in addition to the T4 genes implicated in degradation or inhibition of expression of unmodified (dCMP) DNA (Alc, DenA, and DenB) [20]. Further support that RB69 DNA is likely modified is that it is cleaved by type-IV restriction enzyme GmrSD which does not cleave unmodified DNAs, such as that of the Lambda phage [6]. In addition, restriction of RB69 infection by this and related enzymes is prevented by its internal protein I (IPI*) which is very similar (four residues different) to T4 IPI*, a GmrSD inhibitor [7,21,22]. Based on our previous work on type-IV restriction enzymes and T4 IP proteins, we were motivated to clarify if RB69 hmC DNA underwent hypermodification, and if so what the nature of that modification was.

In this work we demonstrate the existence of a novel epigenetic phage DNA modification, arabinosyl-hmC (ara-hmC), that extends the repertoire of the T-even phage family modifications and suggests the likelihood of other, possibly even more complex, sugar modifications decorating the hmC residues of other phages. We have identified a novel transferase, likely responsible for this arabinosyl-specific modification, and a novel gene module unique to RB69 and its relatives located between the DNA polymerase and UvsX genes that may have a role in the generation of the UDP-arabinose employed by the novel transferase.

## 2. Materials and Methods

### 2.1. Purification of RB69 DNA

High titer stocks of RB69, T4, and T4-related phage DDY1 were propagated from single plaques on *E. coli* BE and purified by CsCl gradient ultra-centrifugation as described by Sambrook et al. [23]. Purified phage particles were disrupted with SDS and proteinase K treatment, and their DNAs extracted using phenol/chloroform and ethanol precipitation with resuspension in sterile water or TE buffer [10 mM Tris-HCl (pH 8.0), 0.1 mM ethylenediaminetetraacetic acid (EDTA)] as described by Sambrook et al. [23].

### 2.2. Biochemical Analyses of RB69 DNA

Hydrolysis reactions were performed using 15 µg of phage DNA diluted to 300 µL in water. An equal volume of 4 M trifluoroacetic acid (TFA) was added and the mixture heated at 100 °C in a heating block for three hours. The samples were cooled to room temperature, lyophilized, and then reconstituted in water (60 µL). The samples were subjected to high-pH anion exchange chromatography with pulsed amperometric detection (HPAED-PAD) analysis in duplicate by injecting a 10-µL sample volume. Standards were also analyzed for identification and quantitation (the signal from 1.0 nmol of glucose gave a peak area of 20.0 and therefore a response of 0.05 nmol/area). Dionex analysis was carried out on an ICS5000 instrument using a Carbopac™ PA10 (4 × 250 mM) (Sunnyvale, CA, USA) analytical column. Samples were eluted using an isocratic 18 mM NaOH solution.

Matrix assisted laser desorption ionization-time of flight mass spectrometry (MALDI-TOF MS) analysis was performed on a Bruker UltrafleXtreme MALDI-TOF/TOF mass spectrometer (Sunnyvale, CA, USA)in positive reflector mode. Thus, 100 mg/mL of 2,5-dihydroxybenzoic acid (Sigma-Aldrich, St. Louis, MO, USA) matrix was prepared in 1:1 H_2_O/acetonitrile solution, with additional 20 μL of *N*,*N*-dimethylaniline (Sigma-Aldrich).

### 2.3. Bioinformatic Analyses of Glucosyltransferases in T4-Related Phages

Nucleotide similarity between the T4 genome and those of related phages, including the sequenced “RB” phages, was determined using Blastn at NCBI. GenBank Identifiers for the currently sequenced “RB” phages are provided in Supplementary Table S1. Dr. James Nolan and Dr. Jim Karam generously provided the T2 and T6 sequences. Homology searches were conducted using a locally implemented version of Psi-Blast and the NCBI non-redundant (nr) plus environmental protein (env_nr) databases [24]. Proteins were inferred to be homologs if the matches typically aligned end to end with the seed protein and their E-values decreased with each iteration. E-values used for the cutoff value for creating families of homologs varied (depending upon where the Psi-Blast search converged) but the highest expect or E-value in this study was 3e-14 (on a third iteration). More diverged homologs to T4 a-gt and other transferase genes were sought using hidden Markov model (HMM)-based searches using local implementations of the Sequence Analysis and Modeling System (SAM [25,26]) and HHPred [27]. HHMs were calibrated against the scop70_1.72pre.hhm library, which with other libraries was downloaded from ftp://toolkit.lmb.uni-muenchen.de/HHsearch/databases/. Protein structures were predicted using Phyre2 (available at http://www.sbg.bio.ic.ac.uk/phyre2/html/page.cgi?id=index) [28].

### 2.4. Cloning of the RB69 ORF053c–ORF052c Gene Region

The 1687-bp genome region spanning RB69 genes ORF053c and ORF052c (genome co-ordinates 28,094 to 29,780, complemented) was amplified using *Pfu* polymerase using forward primer 5′-GCGCCATGGCAAAAGCTGTTATTCTTGGTGCTGGATTAG-3′ and reverse primer 5′-GCGTCTAGATTAGATTCGTTCCCATTGATGAGTGATATCGCC-3′. The resulting product was purified using a QIAquick PCR Purification Kit (Qiagen, Hilden, Germany) and then cloned into the vector pHERD20T using the restriction sites *Nco*I and *Xba*I. Since we planned to express the ORF053c and ORF052c region after cloning into pHERD20T, the second codon of the ORF053c gene was changed from a lysine (AAA) to an alanine (GCA) codon to accommodate the start codon position in this vector being in the *Nco*I site (CCATGG). Constructs were confirmed via Sanger sequencing using the pHERD20T forward and reverse sequencing primers [29].

## 3. Results

### 3.1. HPAEC-PAD and Mass Spectrometry of RB69 DNA Indicates It Contains Arabinose

**Figure 1:**
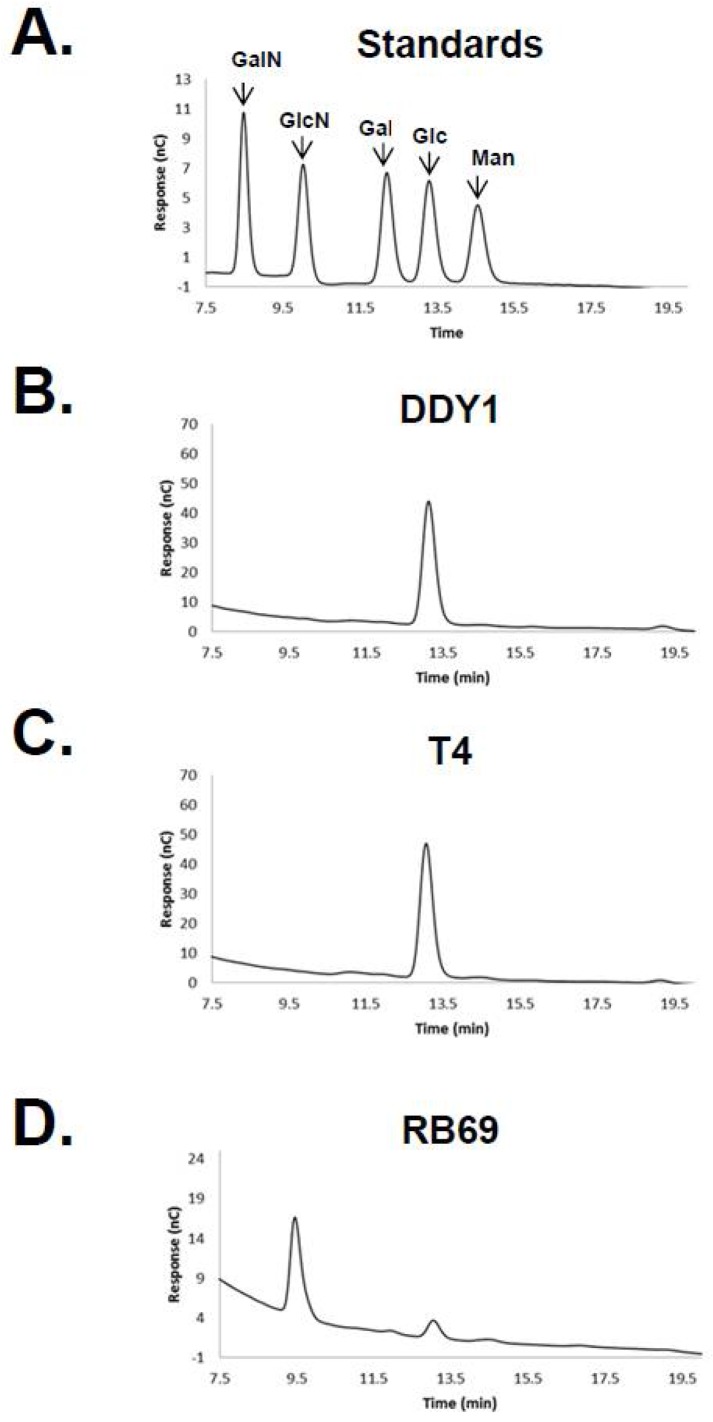
Monosaccharide composition analysis by high-pH anion exchange chromatography with pulsed amperometric detection (HPAEC-PAD). (**A**) monosaccharide standards; (**B**) hydrolysate of DDY1 sample; (**C**), hydrolysate of T4; and (**D**) hydrolysate of RB69 DNA sample. Abbreviations used: GalN, galactosamine; GlcN, glucosamine; Gal, galactose; Glc, glucose; Man, mannose.

Previous studies have demonstrated the accuracy and discrimination of high-pH ion exchange chromatography for the separation of carbohydrates [30], and therefore HPAEC-PAD was employed to analyze the sugar content of RB69, T4, and DDY1 (a T4-related phage) DNAs. Hydrolyzed phage DNAs were analyzed with the monosaccharide, galactosamine, glucosamine, galactose, glucose, and mannose as standards (Figure 1). The T4 and DDY1 samples showed a single major peak which correlated with glucose and were determined to both contain ~0.35 nmole/µg of glucose. The RB69 sample showed two peaks, a minor peak which co-migrated directly with glucose and a major peak which did not co-migrate with any of the standards and represented an unknown sugar. The minor RB69 peak was determined to contain 20-fold less glucose than the T4 and DDY1 samples, the same amount as a water control (15.4% vs. 16%), and this peak was missing from a subsequent sample (see below).

**Figure 2:**
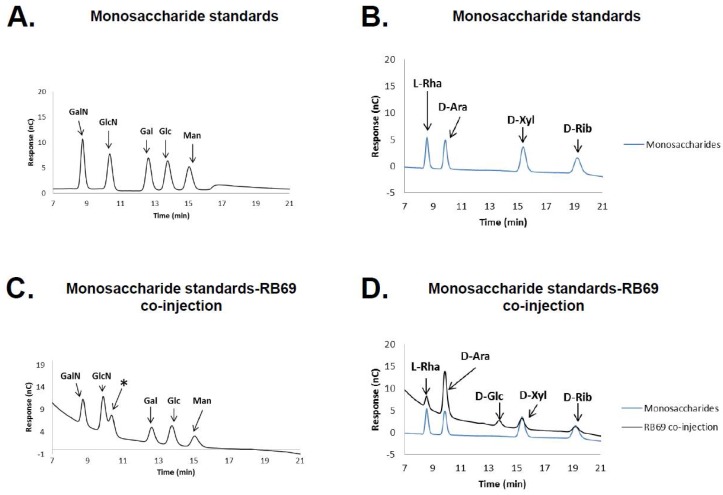
High-pH anion exchange chromatography of monosaccharide standards and RB69 DNA hydrolysate. HPAEC chromatograms of (**A**,**B**) monosaccharide standards and (**C**,**D**) co-injection of monosaccharides with RB69 DNA hydrolysate. Abbreviations used: GalN, galactosamine; GlcN, glucosamine; Gal, galactose; Glc, glucose; Man, mannose; *, Unidentified sugar; l-rha, l-rhamnose; d-Ara, d-arabinose; d-Xyl, d-xylose; d-Rib, d-ribose.

**Figure 3:**
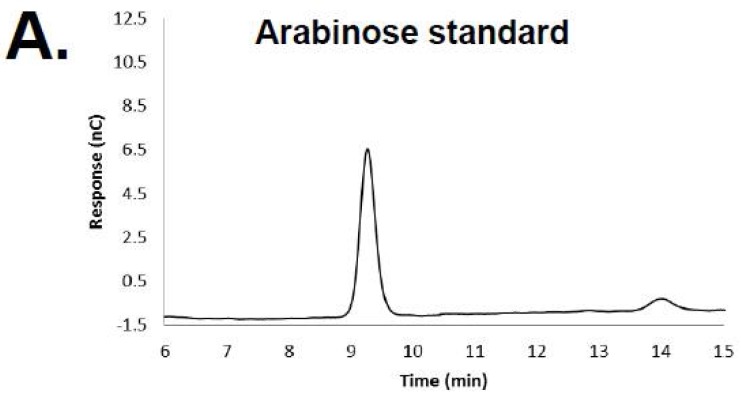

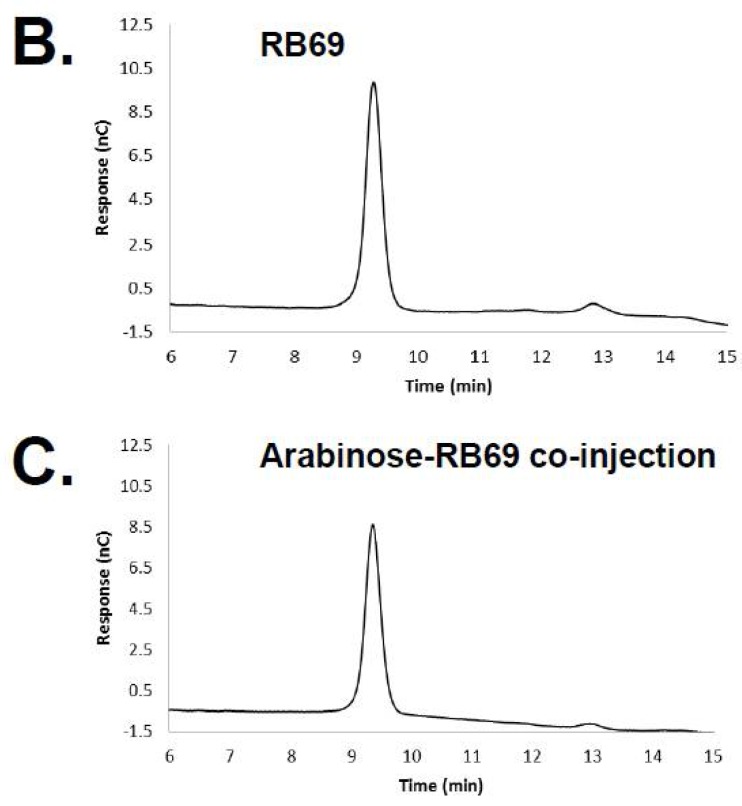
High-pH anion exchange chromatography of arabinose standard and RB69 DNA. HPAEC chromatograms of (**A**) arabinose standard, (**B**) RB69 DNA hydrolysate, and (**C**) co-injection of arabinose standard and sample RB69 DNA hydrolysate.

HPAED-PAD was repeated again on a RB69 DNA hydrolysate including the co-injection of the hydrolyzed RB69 DNA with the monosaccharide controls and the additional monosaccharide standards l-rhamnose, d-arabinose, d-xylose, and d-ribose (Figure 2). Comparison of the hydrolyzed RB69 DNA sample, including its co-injection with the monosaccharide standards, showed that the RB69 peak directly co-migrated with that of d-arabinose, suggesting that RB69 DNA is likely glycosylated with d-arabinose (Figure 2). To confirm this observation, a fresh sample of RB69 DNA underwent hydrolysis and HPAED-PAD repeated with just the arabinose standard, again showing the RB69 peak to directly co-migrate with that from the arabinose standard (Figure 3).

**Figure 4:**
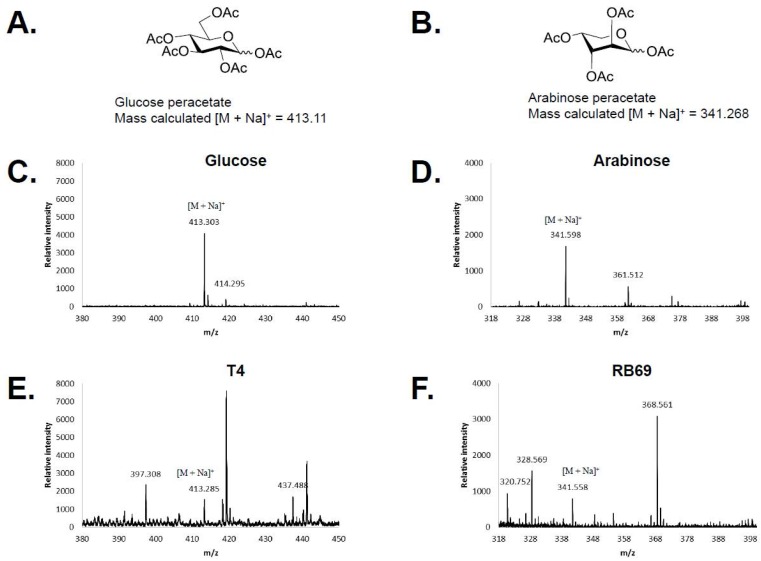
Matrix assisted laser desorption ionization-time of flight (MALDI-TOF) mass spectrometry mass spectrometry analysis of peracetylated samples of T4 and RB69 DNA hydrolysates. (**A**) Structure of glucose peracetate; (**B**) Structure of arabinose peracetate and mass spectrums of (**C**) peracetylated glucose, (**D**) peracetylated arabinose, (**E**) peracetylated T4 DNA hydrolysate, and (**F**) peracetylated RB69 DNA hydrolysate.

The T4 and RB69 DNA hydrolysates were subsequently peracetylated and analyzed by MALDI-TOF mass spectrometry. Comparison of the mass spectra obtained for the phage DNA samples with those from the glucose and arabinose peracetylated standards further confirmed the identification of glucose in the T4 samples and arabinose in the RB69 sample (Figure 4).

### 3.2. Homologs of T-Even Glucosyltranferases in T4-Related and the “RB” Phages

We sought to determine whether RB69 might have a diverged homolog to the characterized T-even glucosyltransferases that was responsible for its ara-hmC modification. We also sought to determine if there were homologs to these enzymes in other “RB” phages. Fifteen of the original 31 “RB” phages [19] have now undergone genome sequencing [20,31] and 14 of these genomes have ≥95% identity (with ≥85% query coverage) to that of T4 as determined by Blastn (Supplementary Table S1). The RB69 genome has less similarity (77% identity, with 70% query coverage) to the T4 genome. BlastP searches from T4 a-gt, T4 b-gt, and T2 ba-gt found no sequence similarity between these enzymes and confirmed there was no identifiable homolog to these enzymes in RB69 using this algorithm. In contrast, all the other sequenced “RB” phages have homologs to two of these enzymes. Twelve “RB” phages have a homolog to T4 a-gt and T2 ba-gt, whereas two phages RB55 and RB59 have homologs to both T4 a-gt and b-gt (Table 1), consistent with their genomes having the highest nucleotide similarity to that of T4 [31] (Supplementary Table S1). Numbers of other phages also have homologs, albeit more diverged, to two glucosyltransferases, such as *Enterobacteria* phage CC31, *Salmonella* phage STML-198, *Citrobacter* phage Moon and *Serratia* phage PS2, which all have a homolog to T4 b-gt and a homolog to T2 ba-gt (Table 1). These observations indicate that, as first hinted by the early studies on T2, T4, and T6, the glucosylation of T4-related phage DNA is likely widespread, and between different phages there may be great variability in the linkages for these modifications.

### 3.3. Identification of RB69 ORF003c as a Putative Arabinosyltransferase

To address the problem of an unidentified glycosyltransferase responsible for the ara-hmC modification in RB69 we decided to apply hidden Markov model (HMM)-based strategies that we have previously employed to identify highly diverged phage proteins (e.g., [32,33,34]). A Psi-Blast search from T4 a-gt (gp59) identified 66 homologs in other phages and prokaryotes. These homologs were aligned and a corresponding HMM made using a local implementation of the Sequence Analysis and Modeling System (SAM [25,26]). Hmmscore (a profile to sequence search) was used to test this custom HMM against libraries of the proteomes of T4, *Bacillus* phage G (which has two diverged homologs to the T4 a-gt, (Table 1)) and RB69. Our logic for this approach was that although these libraries are small, this approach has been demonstrated to be useful for identifying diverged homologs (e.g., [32,33,34]) which will ideally score better than other, non-homologous proteins in the library.

By this strategy RB69 ORF003c (366 residues) was initially indicated as a possible candidate for the sought arabinosyltransferase as it had a similar E-value when searched against the T4 a-gt HMM as obtained for the T4 b-gt (which was not included in the T4 a-gt HMM) (Table 2). We then created an HMM using a larger and more diverse set of homologs obtained from a Psi-Blast search from phage G gp306. Phage G gp306 is annotated as a DNA alpha-glucosyltransferase and conserves a number of residues identified as functionally important in T4 a-gt (see below and Supplementary Figure S1) by Lariviere at al. (2005) [15], but based on its high degree of divergence to T4 a-gt, gp306 requires biochemical analyses to characterize its sugar specificity/activity. However, for the purpose of identifying a diverged glycosyltransferase, phage G gp306 appeared a good candidate to employ for a custom HMM as it has many homologs identifiable by Psi-Blast in both phage and prokaryotic genomes. We aligned 4752 homologs to gp306 and created a corresponding HMM using SAM. The resulting custom Phage G gp306 HMM had improved capability to identify diverged glycosyltransferase homologs using hmmscore (e.g., the E-value obtained for T4 b-gt was approximately seven orders of magnitude lower than that obtained using the custom T4 a-gt HMM, Table 2). The E-value for RB69 ORF003c was similarly improved, as were homologs to ORF003c in other phages such as *E. coli* phage JS09, *Shigella* phage Shfl125875, and *Acinetobacter* phage Acj61 (Table 2) that we identified using BlastP. Notably, another RB69 protein ORF53_52c which we had initially considered as a candidate for a glycosyltransferase (see below) did not score well against either custom HMM (Table 2).

Based on our hmmscore results we converted our phage G gp306 alignment into an HHM using HHpred [27,35] and calibrated it against the scop70_1.72pre.hhm library. We then created a RB69 ORF003c HHM again basing the initial SAM alignment on a homolog set obtained from a Psi-Blast search. This Psi-Blast search identified ten homologs to ORF003c in other phages, such as *E. coli* phage JS09 gp177 and *Shigella* phage Shf125875 gp003 (Supplementary Table S2). Two *Acinetobacter* phages have two homologs to ORF003c; Acj61 (p070 and p076) and Acj9 (p080 and a more diverged homolog split between the two ORFs p082-p081). The ORF003c Psi-Blast search converged after the second iteration, however it did include several weak matches to bacterial proteins annotated as putative glycosyltransferases, such as the putative UDP-galactose—lipooligosaccharide galactosyltransferase of *Haemophilus influenzae* 86-028NP (gb|AAX88754.1, E value of 0.06, 28% identity).

An HHpred search of the ORF003c and phage G gp306 HHMs supported the existence of extremely diverged similarity (10% identity) between the two proteins (Supplementary Figure S2). In spite of this divergence, we noted the alignment of several ORF003c residues with phage G gp306 residues that are conserved to those with functional significance in T4 a-gt (marked in Supplementary Figure S2, see also Supplementary Figure S1). Notably, ORF003c Gly-20 and Arg-207, align with residues in phage G gp306 which are conserved with T4 a-gt Gly-15 and Arg-204 which interact with the beta-phosphate [15]. Importantly, ORF003c Glu-281 is conserved across to T4 a-gt Glu-311 which interacts with ribose and is a residue conserved in all glycosyltransferases of the GT-B class [15]. No residues in T4 a-gt (e.g., His-114, His-116, and His-140) that interact with glucose were conserved across to RB69 ORF003c, consistent with the expectation that ORF003c would have to have different sequence and structural requirements due to its reaction with a different sugar to T4 a-gt.

**Figure 5:**
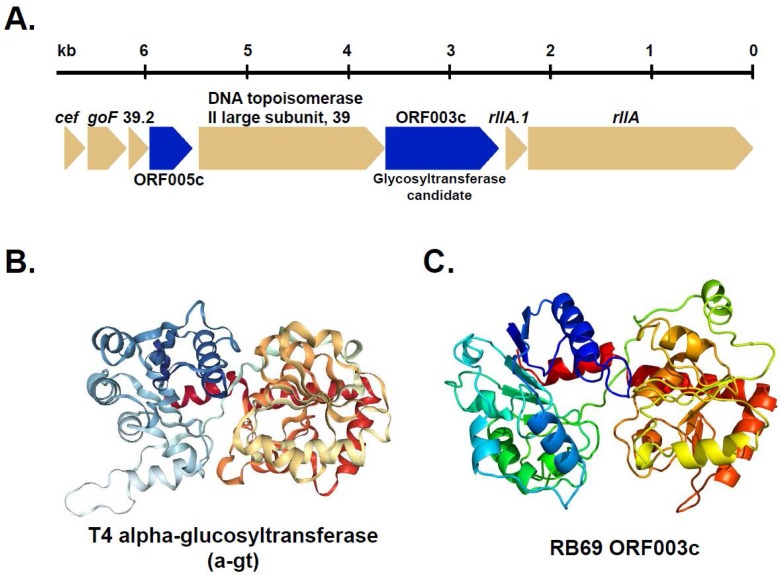
Phage RB69 arabinosyltransferase candidate ORF003c. (**A**) Region of the RB69 genome encoding the putative arabinosyltransferase gene ORF003c. Genes with homologs in T4 are shaded light brown and genes unique to RB69 are shaded dark blue (**B**) Structure of the T4 a-gt (PDB 1XV5) and (**C**) predicted structure of RB69 ORF003c by Phyre2 (intensive mode).

Since these analyses indicated that RB69 ORF003c was a good candidate for the arabinosyl transferase, likely with a GT-B fold, we sought independent evidence of such a function and structure via HHpred searches using the web-based software (available at: https://toolkit.tuebingen.mpg.de) with the database PDB_mmCIF70 selected for modeling. Notably, this search identified an extensive number of glycosyltransferase matches with extremely diverged similarity (≤13% identity) to RB69 ORF003. Supporting the validity of these matches was the low E-value of the matches consistent with those obtained from similar HHpred searches of known phage glucosytransferases against the same proteins (see Table 3 for examples selected as the five top matches to phage G gp306). Similarly, the predicted secondary structure elements for ORF003c HHM were regularly aligned with the structural elements of the GT-B fold, which is essentially two Rossmann-like folds with an active site in a cleft between the two domains [15], in these alignments (e.g., see Supplementary Figures S4–S6). Additionally, a predicted structure for ORF003c by Phyre2 showed overall similarity to the two Rossmann-like folds characteristic of many glycosyltransferases (Figure 5). Although the activity of RB69 ORF003c requires biochemical confirmation, based on our analyses we propose it to be an excellent candidate for the arabinosyltransferase responsible for the final step of ara-hmC production.

### 3.4. Identification of Candidate RB69 Genes for UDP-Arabinose Generation

During our search of the RB69 genome (GenBank identifier NC_004928.1) for a candidate arabinosyltransferase we saw that the *N*-terminal region (residues 4-111) of hypothetical protein RB69ORF053c was commented as having homology to the glycosyltransferase family A superfamily CDD 299700. Although the E-value for this match was promising (1.98e-15), RB69ORF053c was only 221 residues long, which was short relative to the characterized phage glucosytransferases (e.g., T4 a-gt has 400 residues and b-gt has 351 residues). We noted that there were homologs to ORF53c in other phages, such as Acj61 (gp84), Ac42 (gp42), and IME08 (gp43), which had an additional ~300 residues on their *C*-termini relative to ORF053c. BlastP searches from these homologs showed that these *C*-termini regions had similarity to RB69 ORF052c, the gene immediately downstream of RB69053c. Since we observed no evidence of mobile elements in the region between RB69 ORF053c and ORF052c, we hypothesized they might actually represent a single gene, as the RB69 genome was sequenced with Sanger sequencing prior to introduction of high-coverage sequencing now available with next generation sequencing technologies. To test this possibility, we amplified and cloned the 1.69-kb region spanning ORF053c and ORF052c into the expression vector pHERD20T. Sequencing of the cloned fragment found no cytosine corresponding to C-29116 in the GenBank entry, indicating that a single base sequence error had resulted in the classification of separate short genes ORF053c and ORF052c in RB69. With the base change, the entire region is predicted to encode the single open reading frame we designated ORF53_52c (Supplementary Figure S3). Supporting that this region encodes a single 561-residue protein, a crude induction experiment of this RB69 region cloned into pHERD20T produced a single densely stained band by SDS-PAGE that migrated to a position consistent with the predicted molecular weight of ORF53_52c (63.9 kDa) (Supplementary Figure S7). A counterpart to this band was not observed in a pHERD20T-only control.

**Figure 6:**
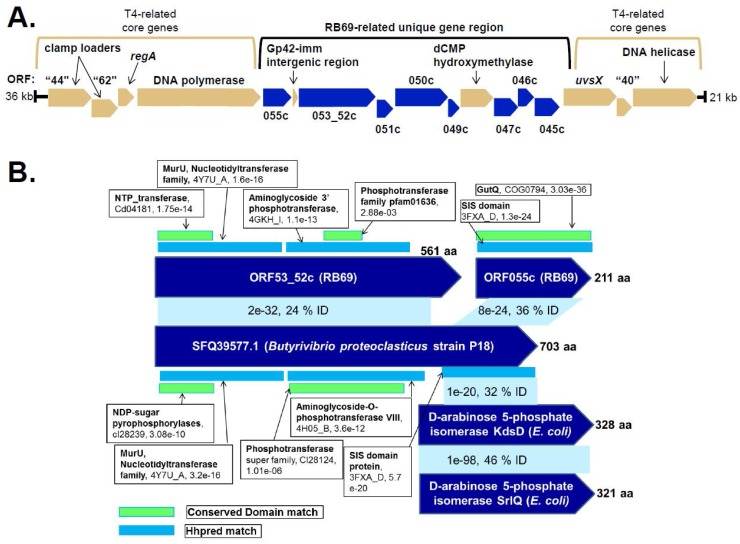
Candidate RB69 genes involved in the synthesis of ara-hmC DNA. (**A**) Region of the RB69 genome encoding novel genes relative to T4. RB69 genes with homologs in T4 are shaded light brown. RB69 genes with no homolog in T4 are shaded dark blue. (**B**) Scheme summarizing the similarity between RB69 ORF53_52c and ORF055c with *Butyrivibrio proteoclasticus* and *E. coli* proteins.

Unexpectedly, when we conducted a search with the full-length ORF53_52c protein we realized this relatively large protein had a complex domain structure; due to this we identified no glycosyltransferase homologs to ORF53_52c by Psi-Blast, and ORF53_52c scored poorly against our phage glucosyltransferase models used to identify ORF003c (Table 2). A BlastP search identified 32 homologs to ORF53_52c in other phages, all of similar length (545–573 residues). These homologs included highly similar matches (≥97% identity) in seven phages (*E. coli* O157 typing phage 3, *Escherichia* phages JS09, APCEc01, phiC120, and phiE142, and *Shigella* phages Shf125875 and SHSML-52-1) and increasingly diverged matches ranging from 73% (*Serratia* phage CHI14) to 24% identity (*Sphingomonas* phage PAU). In addition, there were many highly diverged homologs to ORF53_52c in prokaryotes, e.g., there were 80 matches with ~30% identity to ORF53_52c with E-values of 1.0e-11 or less. Many of the prokaryotic homologs to RB69 ORF53_52c were annotated as hypothetical proteins; however five homologs were annotated as d-arabinose 5-phosphate isomerases GutQ, the best scoring matches being to those of *Butyrivibrio proteoclasticus* strain P18 and *Butyrivibrio* sp. Su6. While these *Butyrivibrio* matches to ORF53_52c are highly diverged (25% and 24% identity, respectively) they had convincing E-values (1e-24 and 2e-24, respectively) and the matches (~500 residues) extended over much of ORF53_52c (Figure 6). HHpred searches of the regions with homology between the *B. proteoclasticus* strain P18 protein (SFQ39577.1) and ORF53_52c showed each to have unusual domain composition with a range of diverged matches, such as to NTP-transferases and pyrophosphotransferases, making functional prediction difficult.

Our searches revealed that the GutQ annotations of the *Butyrivibrio* proteins were likely the consequence of strong matches between their *C*-termini and the conserved cl00389 SIS (Sugar ISomerase) domain (e.g., 5.13e-41 for that of *B. proteoclasticus* strain P18 (SFQ39577.1)). This was also supported by a Blast2seq BlastP match between SFQ39577.1 and the proteome of *E. coli* strain K-12 substr. MG1655 (U00096.3) which identified the two diverged homologs KdsD (1e-20, 32% identity) and SrlQ (2e-15, 31% identity) over ~170 residues on the *C*-terminus of SFQ39577.1. KdsD and SrlQ are known paralogs and both are annotated as d-arabinose 5-phosphate isomerases based on the biochemical analyses of SrlQ, also known as GutQ, which was shown to catalyze the reversible aldol-ketol isomerization between d-ribulose 5-phosphate and d-arabinose 5-phosphate [36,37]. As the match between the *B. proteoclasticus* SFQ39577.1 protein and ORF53_52c did not extend to this *C*-terminal SIS-like domain, we sought a counterpart to this region in RB69. A BlastP search identified the product of a gene upstream to ORF53_52C, the 211 residue ORF055c, as a homolog to this domain (Figure 6). Based on the divergence of the matches between the *B. proteoclasticus* protein SFQ39577.1, and its characterized *E. coli* homolog, as well as its counterparts in RB69, further research is required to clarify the functions of these novel proteins. However, based on the fact that RB69 ORF055c and ORF53_52 have domain matches that link them back to an enzyme that recognizes arabinose, and that RB69 has a confirmed ara-hmC modification, we speculate that these proteins are excellent candidates for having a role in the generation of UDP-arabinose that would be required for the formation of ara-hmC. In addition, the genes encoding these RB69 proteins are in a gene module containing eight genes that have no homologs in T4, located between the DNA polymerase and UvsX genes (Figure 6)—a region shown to have a high degree of plasticity in T4 phages. For instance, the T4 b-gt and homing nuclease SegA genes are located between its dCMP hydroxymethyltransferase and UvsX genes. Notably, several phages with homologs to the putative RB69 arabinosyltransferase ORF003c, such as *E. coli* phage JS09 and *Shigella* phage Shf125875, have counterparts to the RB69 gene region that includes the highly unusual ORF055c and ORF53_52c genes that may be involved in UDP-arabinose synthesis.

## 4. Discussion

In this work we demonstrate the existence of a novel phage epigenetic DNA modification, arabinosyl-hmC, in the RB69 phage that extends the DNA modification repertoire of the T-even phage family. Chemical analysis of RB69 DNA shows it to contain arabinose rather than glucose as found in the T4-type phage or related phage DDYI, although the extent of the hmC arabinosylation is uncertain. In this study we also identified RB69 ORF003c as a strong candidate for the novel arabiosyltransferase responsible for the final step of the ara-hmC modification. RB69 ORF003c likely has a GT-B fold, as observed for the structurally analyzed a-bt and b-gt enzymes of the T4 phage. Based on the existence of close homologs to ORF003c in other phages, including *E. coli* phage JS09 and *Shigella* phage Shf125875 (for which homologs have a slightly higher similarity at the sequence level to ORF003c than that between T2 and T4 a-gts) it seems likely that the ara-hmC modification exists in other T4-related phage DNAs. In addition, our analyses indicate that hidden Markov model-based approaches could be useful for the identification of further novel transferase genes in other phages. Such studies are important as hmC has recently reemerged as being of considerable interest as an epigenetic modification in eukaryotes. Intriguingly, phage glucosyltransferases have been shown using bioinformatics to extend potentially into the human genome [38].

Notably, previous studies have shown that T4, DDY1, and RB69 DNAs are subject to restriction by the GmrSD family of type-IV enzymes [21,39]. These enzymes were thought to target hmC or glucosyl-hmC DNA, yet this study indicates that the specificity of the CT596 GmrSD enzyme must extend to arabinosyl-hmC DNA. However, the type of glycosyl modification may impact their activity, as the UTI89 GmrSD enzyme is unable to restrict T4 or DDY1 DNA but can restrict RB69 DNA. It should be noted that this specificity may be complicated by an additional factor. Phages in the T-even family package in their capsids a diverse family of protein inhibitors of type-IV REs to which they are susceptible. The T4 IPI, or internal protein I, is particularly effective in blocking the nuclease of the type-IV RE CT596 from inactivating phage T4, but is not effective in blocking the closely homologous single chain UTI89 RE [6,7,21]. However, the RB69 IPI protein has four residues that are different to those of T4 IPI, so whether it is this difference or the ara-hmC modification that makes RB69 susceptible to attack by the UTI89 enzyme requires clarification. There is expected to be a high degree of impact from the different sugar modifications as well as possibly of the phage-injected protein inhibitors on different type IV REs as well as on CRISPR-Cas-directed Res, as recently shown by Bryson et al. [8].

We note that arabinosylation of the RB69 hmC residues logically requires the synthesis of UDP-arabinose as compared to the *E. coli*-generated UDP-glucose required by both T4 a-gt and b-gt. This represents an unprecedented biochemical scenario with regard to phage DNA modification and at this stage the biochemistry for the production of UDP-arabinose is unclear; there are several possible pathways. For instance, UDP-arabinose might be synthesized via an UDP-xylose intermediate, as recently demonstrated in *Sinorhizobium meliloti* 1021 SMb20458 [40]. In *S. meliloti* the enzyme Uxs, a UDP-xylose synthase, decarboxylates UDP-glucuronic acid to UDP-xylose, and Uxe, a UDP-xylose 4-epimerase, interconverts UDP-xylose and UDP-arabinose [40]. No homologs to these enzymes have been identified in *E. coli* to our knowledge, so presumably if a similar pathway was utilized for the UDP-arabinose employed for the RB69 ara-hmC modification, phage-encoded enzymes would be required.

Alternatively, a combination of host- and phage-derived enzymes might be required to produce the UDP-arabinose precursor. For instance, host-derived 4-amino-4-deoxy-l-arabinose (l-Ara4N), as described by Trent et al. [41], might be converted to UDP-arabinose by phage-encoded enzymes. Alternatively, conversion of arabinose-1-PO_4_ to arabinose-5-PO_4,_ and vice versa might facilitate UDP-arabinose production by phage-encoded enzymes (e.g., a kinase and esterase). Resolving the mechanism for the generation of UDP-arabinose required for the RB69 arabinosyl modification represents an exciting opportunity for future research. Based on the RB69 genes with similarity to proteins known to manipulate sugars we identified in this study we speculate RB69 is indeed very “odd” and has a role in the synthesis of UDP-arabinose.

Intriguingly, RB69 was shown to uniquely to exclude its DNA from genetic exchange with 33 other T-even-related hmC-related phages (T4, T2, T6, and the other “TB” phages), and as a result this defining characteristic was described as forming a discrete species [19]. The sequencing of the RB69 genome revealed considerable divergence in the nucleotide sequences of many of its counterparts to T4 genes, but it was noted that the sequence divergence between the two genomes did not explain the lack of recombination between the two genomes [20]. This study has demonstrated that, with the exception of RB69, the other 14 sequenced “RB” phages have DNAs that are likely similarly glucosylated to those of T2, T4 or T6. This leads us to speculate that the different sugar modification of RB69 DNA contributed to its inability to recombine with the other RB-phages. If ara-hmC was the basis for the described RB69 speciation, this would support a recent bold proposal that DNA modification is the epigenetic basis for transposon-derived speciation in eukaryotes [42].

While further research is required to understand the biochemistry behind ara-hmC formation and its function(s), the identification of this novel DNA sugar modification in this study in a relatively well studied phage highlights the likelihood of further novel phage DNA sugar modifications yet to be identified. The identification of ara-hmC also opens the possibility of the presence of ara-hmC in other organisms. Our finding indicates that the evolutionary arms race between bacterial restriction enzymes and phage DNA modifications is likely even more complex than previously realized.

## 5. Conclusions

In this work we demonstrate the existence of a novel phage epigenetic DNA modification, arabinosyl-hmC (ara-hmC) using HPEAC and mass spectrometric analyses. This ara-hmC discovery extends the repertoire of the T-even phage family and opens up the possibility of other, even more complex sugar modifications decorating hmC residues. It appears that ara-hmC is the basis for a long-standing puzzle [19,20]. For unknown reasons RB69 was shown uniquely to be excluded from co-infection and from recombination-based genetic exchange with other T-even and RB phages containing g-hmC, the latter a defining criterion for speciation. This research opens the door to exciting further studies on what types of sugar and other modifications exist in the DNAs of phages and other organisms and the impact of these modifications on their ability to undergo genetic exchange. In addition, we have identified a novel transferase in RB69 as well as candidate genes involved in UDP-arabinose formation, all of which are excellent targets for further research to determine the biochemistry behind this new arabinosyl modification.

## Figures and Tables

**Table 1:** Homologs to the T4 and T2 glucosyltransferases in sequenced “RB” phages as determined by BlastP. n/a refers to not applicable as no homolog was detected.

Phage	T4 a-gt (400 aa)		T4 b-gt (351 aa)		T2 ba-gt (280 aa)	
	Protein Accession	Match Identity, %	Protein Accession	Match Identity, %	Protein Accession/Name	Match Identity, %
T4	NP_049673.1	100	NP_049658.1	100	n/a	n/a
T2 ^1^	a-gt_T2	91	n/a	n/a	Q06717.1	100
T6 ^1^	a-gt_T6	99	n/a	n/a	Q06718.1	98
RB3	YP_009098445	91	n/a	n/a	YP_009098428.1	99
RB5	AIT73068.1	91	n/a	n/a	AIT73051.1	99
RB6	AIT73339.1	91	n/a	n/a	AIT73322.1	99
RB7	AIT73610.1	91	n/a	n/a	AIT73593.1	99
RB9	AIT73882.1	91	n/a	n/a	AIT73865.1	99
RB10	AIT74154.1	91	n/a	n/a	AIT74137.1	99
RB14	YP_002854395.1	92	n/a	n/a	YP_002854377.1	99
RB27	YP_009102266.1	91	n/a	n/a	YP_009102248.1	99
RB32	YP_803002.1	91	n/a	n/a	YP_802983.1	98
RB33	AIT74699.1	91	n/a	n/a	AIT74680.1	98
RB51	YP_002854018.1	91	n/a	n/a	YP_002853998.1	99
RB55	AIT74973.1	100	AIT74956.1	100	n/a	n/a
RB59	AIT75245.1	100	AIT75228.1	100	n/a	n/a
RB68	YP_009167432.1	91	n/a	n/a	YP_009167412.1	99
RB69	n/a	n/a	n/a	n/a	n/a	n/a
Examples of homologs in other T4-related phages						
*Yersinia* phage PST	YP_009153660.1	99	n/a	n/a	YP_009153643.1	97
*Shigella* phage Shfl2	YP_004414960.1	91	n/a	n/a	YP_004414944.1	99
*Escherichia* phage wV7	YP_007004802.1	91	n/a	n/a	YP_007004785.1	99
*Escherichia* phage AR1	YP_009167872.1	91	n/a	n/a	YP_009167855.1	99
*Escherichia* phage HY01	YP_009148507.1	91	n/a	n/a	YP_009148490.1	98
*E. coli* phage e11/2 (slur02)	YP_009210250.1	91	n/a	n/a	YP_009210234.1	99
*E. coli* ACG-C40	YP_006986613.1	94	n/a	n/a	YP_006986593.1	98
Examples of more highly diverged homologs in other phages						
*Enterobacteria* phage CC31	n/a	n/a	YP_004009897.1	49	YP_004009898.1	65
*Enterobacter* phage PG7	n/a	n/a	YP_009005302.1	49	YP_009005303.1	65
*Salmonella* phage S16	n/a	n/a	YP_007501076.1	50	YP_007501077.1	64
*Salmonella* phage STML-198	n/a	n/a	YP_009148028.1	50	YP_009148029.1	64
*Citrobacter* phage Moon	n/a	n/a	YP_009146477.1	50	YP_009146478.1	64
*Serratia* phage PS2	n/a	n/a	YP_009030087.1	53	YP_009030088.1	48
*Salmonella* phage STP4-a	n/a	n/a	YP_009126243.1	50	YP_009126244.1	64
*Citrobacter* phage Merlin	n/a	n/a	YP_009203756.1	48	YP_009203757.1	64
*Bacillus* phage G	YP_009015609.1 (gp306)	24	n/a	n/a	n/a	n/a
*Bacillus* phage G	YP_009015609.1 (gp313)	24	n/a	n/a	n/a	n/a

^1^ T2 and T6 a-gt sequences were obtained from Dr. James Nolan and Dr. Jim Karam.

**Table 2:** Identification of candidate glucosyltransferases in phages RB69, JS09, Shfl125875, and Acj61. Scores for phage proteins were obtained using hmmscore searches with hidden Markov models (HMMs) of either the T4 a-gt or its diverged homolog in *Bacillus* phage G, gp306. *, indicates that a protein was incorporated into the HMM.

Phage, Protein	Protein Accession	E-Value for Protein When Scored Against ^1^	
		T4 a-gt HMM	Phage G gp306 HMM
T4 a-gt	NP_049673.1	3.06e-168 *	3.62e-38 *
T4 b-gt	NP_049658.1	1.07e-06	1.46e-13
Phage G, gp306	YP_009015609.1	9.92e-133 *	6.19e-46 *
Phage G, gp313	YP_009015609.1	1.16e-132 *	5.30e-45 *
RB69, RB69ORF003c	NP_861693.1	3.24e-05	4.61e-19
RB69, ORF052_53c	Supplementary Figure S3	1.82e-02 9.12e+01 ^2^	9.85e-01 2.07e+02 ^2^
JS09, JS09_0177	YP_009037500.1	5.45e-05	2.32e-18
Shfl125875, BI097_gp055	YP_009289016	4.17e+00	1.50e-16
Acj61, Acj61p077	YP_004009694.1	3.50e+00	3.37e-11
Acj61, Acj61p076	YP_004009693.1	11.82e-04	2.90e-15

^1^ scores obtained using the hmmscore sw = 0, sequence-model (global) setting. ^2^ score obtained using the hmmscore sw = 3, subsequence-model (domain) setting due to the longer length of RB69 ORF53_52c.

**Table 3:** HHpred matches to glycosyltransferases by T4 a-gt, T4 b-gt, phage G gp306 and RB69 ORF003c. All matches had a probability of ≥98.1%.

Organism	PDB ID	Glycosyltransferase	E-Value for Phage Protein			
			T4 a-gt	T4 b-gt	Phage G gp306	RB69 ORF003c
T4 phage	1XV5_A	DNA alpha-glucosyltransferase	2.10e-39	3.10e-20	5.30e-33	1.90e-24
*Chlorella* virus NY2A	3OY2_A	Glycosyltransferase B736L; GDP-mannose, GT4	1.60e-35	3.60e-21	4.70e-32	4.00e-26
*Halothermothrix*	2R60_A	glycosyl transferase, group 1	3.30e-35	8.30e-22	8.90e-34	5.60e-26
*Campylobacter*	6EJI_A	WlaC protein; glycosyltransferase	4.90e-34	2.70e-22	1.80e-31	2.90e-27
*Geobacillus*	2X0D_A	WSAF; GT4 family, transferase	1.80e-33	3.20e-22	1.30e-31	4.90e-26

## Supplementary Materials

Supplementary materials can be found at http://www.mdpi.com/1999-4915/10/6/313/s1.
