# Supplementary material for: The Odd “RB” Phage—Identification of Arabinosylation as a New Epigenetic Modification of DNA in T4-Like Phage RB69
Source: Viruses. 2018 Jun 8;10(6):313. doi: 10.3390/v10060313 (PMC6024577; doi:10.3390/v10060313)

Supplementary Table and Figures

“The odd “RB” phage – Identification of arabinosylation as a new epigenetic modification of DNA”

Julie A. Thomas, Jared Orwenyo, Lai-Xi Wang, and Lindsay W. Black

Supplementary Table S1. Genome similarity between T4 phage, "RB" phages and other phages with identified glucosyltransferases (a-gt, b-gt or ba-gt proteins listed in Table 1) as determined by Blastn. NIS, indicates no identifiable similarity by Blastn.

**Phage**	**Query cover, %**	**E value**	**Identity, %**	**Accession**
T4	100	0.0	100	AF158101.6
T2[1]	87	0.0	98	
T6[1]	89	0.0	97	
Enterobacteria phage RB3	87	0.0	97	KM606994.1
Enterobacteria phage RB5	87	0.0	97	KM606995.1
Enterobacteria phage RB6	87	0.0	97	KM606996.1
Enterobacteria phage RB7	87	0.0	97	KM606997.1
Enterobacteria phage RB9	87	0.0	97	KM606998.1
Enterobacteria phage RB10	87	0.0	97	KM606999.1
Enterobacteria phage RB14	86	0.0	95	FJ839692.1
Enterobacteria phage RB27	86	0.0	96	KM607000.1
Bacteriophage RB32	86	0.0	95	DQ904452.1
Enterobacteria phage RB33	86	0.0	95	KM607001.1
Enterobacteria phage RB51	85	0.0	95	FJ839693.1
Enterobacteria phage RB55	99	0.0	99	KM607002.1
Enterobacteria phage RB59	99	0.0	99	KM607003.1
Enterobacteria phage RB68	85	0.0	95	KM607004.1
Enterobacteria phage RB69	77	0.0	70	NC_004928
Other T4-related phages with glucosyltransferase homologs				
Yersinia phage PST	88	0.0	97	KF208315.1
Shigella phage Shfl2	87	0.0	96	HM035025.1
Escherichia phage wV7	86	0.0	95	HM997020.1
Enterobacteria phage AR1	87	0.0	95	AP011113.1
Escherichia phage HY01	87	0.0	95	KF925357.1
Escherichia phage e11/2	89	0.0	97	KJ668714.2
Enterobacteria phage ACG-C40	87	0.0	96	JN986846.1
Other phages with diverged glucosyltransferase homologs				
Enterobacteria phage CC31	52	0.0	72	NC_014662.1
Enterobacter phage PG7	54	0.0	72	NC_023561.1
Salmonella phage S16	56	0.0	72	NC_020416.1
Salmonella phage STML-198	56	0.0	72	NC_027344.1
Citrobacter phage Moon	54	0.0	72	NC_027331.1
Serratia phage PS2	25	0.0	68	NC_024121.1
Salmonella phage STP4-a	57	0.0	72	NC_026607.2
Citrobacter phage Merlin	55	0.0	73	NC_028857.1
Bacillus virus G	2	5e-07	69	NC_023719.1

[1] T2 and T6 nucleotide sequences were obtained from Dr. James Nolan and Dr. Jim Karam

Supplementary Table 2. Homologs to RB69 ORF003C (NP_861693.1) in other phages identified using Psi-Blast.

Phage	Protein name	Protein accession	Query coverage, %	Identity, %	E value
Escherichia phage vB_EcoM_JS09	hypothetical protein JS09_0177	YP_009037500.1	99	100	0.00
Shigella phage SHSML-52-1	hypothetical protein	YP_009289016.1	99	100	0.00
Escherichia phage APCEc01	hypothetical protein APCEc01_026	YP_009224986.1	99	100	0.00
Escherichia phage phiE142	hypothetical protein phiE142_23	ALY07828.1	99	100	0.00
Escherichia phage vB_EcoM_PhAPEC2	hypothetical protein PhAPEC2_3	YP_009056595.1	99	100	0.00
Shigella phage Shf125875	hypothetical protein	YP_009100545.1	99	99	0.00
Escherichia phage ST0	hypothetical protein	ASD53821.1	98	100	0.00
Acinetobacter phage Acj61	hypothetical protein Acj61p077	YP_004009694.1	41	99	9.00E-99
Acinetobacter phage Acj61	hypothetical protein Acj61p076	YP_004009693.1	42	98	3.00E-94
Acinetobacter phage Acj9	hypothetical protein Acj9p080	YP_004010217.1	41	98	8.00E-85
Acinetobacter phage Acj9	hypothetical protein Acj9p081	YP_004010218.1	52	35	6.00E-36
Acinetobacter phage Acj9	hypothetical protein Acj9p082	YP_004010219.1	35	50	4.00E-29

Supplementary Figure S1. Alignment of T4 a-gt (gp59) with homologs in related T4-like phages and phage G (gp306 and gp313). The *N*-terminal domain of T4 a-gt includes residues 1-177 and the *C*-terminal domain residues 176-386 (shaded blue). T4 a-gt residues with important interactions by Lariviere et al., 2005 (JMB 352:139-150)(Table 1) are highlighted. Structural elements from 1xv5_A (T4 a-gt) were added manually to the alignment (S, beta sheet; H, helix).

```
              S1                 H1             S2                       S3          H2         S4                H3
dssp       CEEEEEETTCCCSSHHHHHHHHHHHHHHHTTCEEEEEEEECSSCCTTTTSSSCTTTCEEECTTTTCHHHHHHHHTSCSEEEEEEECCBTTSCHHHHHHHHHH
T4__       MRICIFMARGLEGCGVTKFSLEQRDWFIKNGHEVTLVYAKDKSFTRTSSHDHKSFSIPVILAKEYDKALKLVNDCDILIINSVPATSVQEATINNYKKL
RB14       MKVCIFMARGLEGCGVTKFSLEQRDWFIKNGHEVTLVYAKDKSFTRNCAHDYKSFSIPVLLAKEYDKTLKLVNDCDILIINSVPATSVEEDTINNYKKI
RB32       MKVCIFMARGLEGCGVTKFSLEQRDWFIKNGHEVTLVYAKDKSFTRNCAHDYKSFSIPVLLAKEYDKTLKLVNDCDILIINSVPATSVEEDTINNYKKI
AR1_       MKVCIFMARGLEGCGVTKFSLEQRDWFIKNGHEVTLVYAKDKSFTRNCAHDYKSFSIPVLLAKEYDKTLKLVNDCDILIINSVPATSVEEDTINNYKKI
RB51       MKVCIFMARGLEGCGVTKFSLEQRDWFIKNGHEVTLVYAKDKSFTRNCAHDYKSFSIPVLLAKEYDKTLKLVNDCDILIINSVPATSVEEDTINNYKKI
AR1_       -------------CGVTKFSLEQRDWFIKNGHEVTLVYAKDKSFTRNCAHDYKSFSIPVLLAKEYDKTLKLVNDCDILIINSVPATSVEEDTINNYKKI
T2__       MKVCIFMARGLEGCGVTKFSLEQRDWFIKNGHEVTLVYAKDKSFTRNCAHDYKSFSIPVLLAKEYDKTLKLVNDCDILIINSVPATSVEEDTINNYKKI
T6__       MKVCIFMARGLEGCGVTKFSLEQRDWFIKNGHEVTLVYAKDKSFTRTSSHDHKSFSIPVILAKEYDKALKLINDCDILIINSVPATSVQEATINNYKKL
G306       -KIALIMGQSIEGCGVTRNAAEMFQWSKKAGVKFDIYSYDERMYNRRDAHEMDFISFT---RENINSTVEKLNQYDIVMFNSYPSNKFEKQAIIDFYEK
G313       -KLALIFGLSIEGAGATRNGSEMQHWCDKNGVQFKIFSYDESKFTREESHK---ISYTKFNKYNLKEVVDELNTYDIVMFNTYPFPKVGQEAYENFYHN
```

T4 Gly-15 interacts beta phosphate

T4 Arg-46 interacts with base

```
                     S5                  H4    S6                     H5                     S7       H6              S8
dssp       HHHSCTTSEEEEEEECCCSHHHHTTBSSHHHHHHHCSEEEESCTTSHHHHTHHHHHSCSSCCSSSCCCCCCCEEECCCCBCHHHHHHHHCCCGGGCEEEE
T4         LDNIKPSIRVVVYQHDHSVLSLRRNLGLEETVRRADVIFSHSDNGDFNKVLMKEWYPETVSLFDDIEEAPTVYNFQPPMDIVKVRSTYWKDVSEINMNI
RB14       IDNIKPSVRVVVYQHDHSSLSLRRNLGLEETVRRADVIFSHSDNGDFNKVLMKEWYPETVSLFDDIEEAPTVYNFQPPMDIAKVRSTYWKDVSEINMNI
AR1        IDNIKPSVRVVVYQHDHSSLSLRRNLGLEETVRRADVIFSHSDNGDFNKVLMKEWYPETVSLFDDIEEAPTVYNFQPPMDIAKVRSTYWKDVSEINMNV
RB32       IDNIKPSVRVVVYQHDHSSLSLRRNLGLEETVRRADVIFSHSDNGDFNKVLMKEWYPETVSLFDDIEEAPTVYNFQPPMDIAKVRSTYWKDVSEINMNI
RB51       IDNIKPSVRVVVYQHDHSSLSLRRNLGLEETVRRADVIFSHSDNGDFNKVLMKEWYPETVSLFDDIEEAPTVYNFQPPMDIAKVRSTYWKDVSEINMNI
AR1        IDNIKPSVRVVVYQHDHSSLSLRRNLGLEETLRRSDVIFSHSDNGDFNKVLMKVWYPETVSLFDDIEEAPTVYNFQPPMDIAKVRSTYWKDVSEINMNV
T2         IDNIKPSVRVVVYQHDHSSLSLRRNLGLEETVRRADVIFSHSDNGDFNKVLMKEWYPETVSLFDDIEEAPTVYNFQPPMDIAKVRSTYWKDVSEINMNI
T6         LDNIKPSIRVVVYQHDHSVLSLRRNLGLEETVRRADVIFSHSDNGDFNKVLMKEWYPETVSLFDDIEEAPTVYNFQPPMDIAKVRSTYWKDVSEINMNI
G306       VIKGVTTIK-VGFMHELNKTNIDKIPYLVGIMNEMDVIYNFGEETWFSQTI--------SDLLPSKEIGKRTKKFTMWFNFEDLENNYRNKYSLDDKSK
G313       FVKKITALK-VGFMHEINKVIIDKIPYILGLMNEMDMIYTFGVDTWFSK--------NTSTLLPSKKPNERIKKFTMWFNFELLEEYRKTIQLDDKEKK
```

T4 His114, His116 and His140 interact with glucose

Supplementary Fig. S1 cont.

```
            S8               H7              S9                  S10                S11       H8        S12
dssp       EE.EECCSCGGGCHHHHHHHHHHT.TTTTTCEEEEECCCCSHHHHHHHHHTTCCEEEECGGGGGGCCCSSSCCEEEESCCCHHHHHHHHHTEEEEEECCC
T4         NR.WIGRTTTWKGFYQMFDFHEKF.LKPAGKSTVMEGLERSPAFIAIKEKGIPYEYYGNREIDKMNLAPNQPAQILDCYINSEMLERMSKSGFGYQLSK
RB14       NR.WIGRTTTWKGFYQMFDFHEKH.LKPAGLSTIMEGLERSPAFIPIKEKGIPYEYYRLHQVDQIKIAPNLPTQILDRYVNSEMLERMSKSGFGYQLSK
AR1        NR.WIGRTTTWKGFYQMFDFHEKH.LKPAGLSTIMEGLERSPAFIPIKEKGIPYEYYRLHQVDQIKIAPNLPTQILDRYVNSEMLERMSKSGFGYQLSK
RB32       NR.WIGRTTTWKGFYQMFDFHEKH.LKPAGLSTIMEGLERSPAFIPIKEKGIPYEYYRLHQVDQIKIAPNLPTQILDRYVNSEMLERMSKSGFGYQLSK
RB51       NR.WIGRTTTWKGFYQMFDFHEKH.LKPAGLSTIMEGLERSPAFIPIKEKGIPYEYYRLHQVDQIKIAPNLPTQILDRYVNSEMLERMSKSGFGYQLSK
AR1        NR.WIGRTTTWKGFYQMFDFHEKH.LKPAGLSTIMEGLERSPAFIPIKEKGIPYEYYRLHQVDQIKIAPNLPTQILDRYVNSEMLERMSKSGFGYQLSK
T2         NR.WIGRTTTWKGFYQMFDFHEKH.LKPAGLSTIMEGLERSPAFIPIKEKGIPYEYYRLHQVDQIKIAPNLPTQILDRYVNSEMLERMSKSGFGYQLSK
T6         NR.WIGRTTTWKGFYQMFDFHEKF.LKPAGKSTVMEGLERSPAFIAIKEKGIPYEYYGNREIDKMNLAPNQPAQILDCYINSEMLERMSKSGFGYQLSK
G306       KLvYCSRWTTMKGPRRVLDLAPMLaVKDPEFKAELKGIERS---IGAKFDIFDHPNTLDCTGRTPDPNSTGTVPVYGPYIREEGIDFMAKNLFIASFYR
G313       ML.YLGRFVSTKDVPRVLNLGAEI.LKK--DSNFKTIIRGIDTSIGAKVSVLDHPNAINYIPKEPKVNSNGCVPVYGPYVRDEGMNEMAHSLFGVSFWN
                 T4 Arg204 and Lys209                                                  T4 Tyr275 and Asn277,
                 interact with beta phosphate                                         interact with ribose
                                                                                  Tyr275 also interacts with base
```

```
                             H9      S13         S14  S15       S16         H10         H11                H12
dssp       CCGGGCSS...CCCHHHHHHHHHTSEEEEEHHHHHHSBCTTTCCBGGGSCCSCEEECTTCHHHHHHHHHHHHTCHHHHHHHHHHHHHHHHHHHHHHBHHHH
T4         LNQKYLQR...SLEYTHLELGACGTIPVFWKSTGENLKFRVDNTPLTSHDSGIIWFDENDMESTFERIKELSSDRALYDREREKAYEFLYQHQDSSFCF
RB14       LDKKYLQR...SLEYTHLELGACGTIPVFWKSTGENLKFRVDNTPLTSHDSGIIWFDENDMESTFERIKELSSDRALYDREREKAYEFLYQHQDSSFCF
AR1        LDKKYLQR...SLEYTHLELGACGTIPVFWKSTGENLKFRVDNTPLTSHDSGIIWFDENDMESTFERIKELSSDRALYDREREKAYEFLYQHQDSSFCF
RB32       LDKKYLQR...SLEYTHLELGACGTIPVFWKSTGDNLKFRVDNTPLTSHDSGIIWFDENDMESTFERIKELSSDRALYDREREKAYEFLYQHQDSSFCF
RB51       LDKKYLQR...SLEYTHLELGACGTIPVFWKSTGENLKFRVDNTPLTSHDSGIIWFDENDMESTFERIKELSSDRTLYDREREKAYEFLYQHQDSSFCF
AR1        LDKKYLQR...SLEYTHLELGACGTIPVFWKSTGENLKFRVDNTPLTSHDSGIIWFDENDMESTFERIKELSSAR------------------------
T2         LDKKYLQR...SLEYTHLELGACGTIPVFWKSTGDNLKFRVDNTPLTSHDSGIIWFDENDMESTFERIKELSSDRTLYDREREKAYEFLYQHQDSSFCF
T6         LNQKYLQR...SLEYTHLELGACGTIPVFWKSTGENLKFRVDNTPLTSHDSGIIWFDENDMESTFERIKELSSDRALYDREREKAYEFLYQHQDSSFCF
G306       MPKAIQDYgd.RMEYSQIESIAVGSIPVFDTNWGENNR-TLDGQRYIDVPYSAIYSDEKDLEGTVEKLIEVANNKELQEKYRETSYKIAKQEFDANIVL
G313       MQKRGIEEygdRMEYTQIETIAVGTIPVFDKHWGENNR-TLDGRRYIDIPYSAIYCDRENLNETVDKLIEVANNKELQEKYRNTSYNLAKQEFDASIVL
                   T4 Tyr307 Glu-311-interacts with ribose and conserved in all GT-B glycosyltransferases
                   and Thr308-both interact with alpha phosphate
```

```
              H12
dssp       HHHHHHHTC
T4         KEQFDIITK
RB14       KEQFDIITK
AR1        KEQFDIITK
RB32       KEQFDIITK
RB51       KEQFDIITK
AR1        ---------
T2         KEQFDIITK
T6         KEQFDIITK
G306       PKMFNEI--
G313       PKMFNE---
```

Supplementary Figure S2. HHpred alignment of phage G gp306 (AE093565.1) and RB69 ORF003c (32350306) HHMs. Residues in phage G gp306 that are conserved with residues of known functional importance in T4 a-gt (see Supplementary Figure 1) are shaded yellow and residues which align with them in RB69 ORF003c (or an appropriate residue close by) are also shaded yellow.

```
>gi|32350306|gb|AAP75905.1| RB69ORF003c hypothetical protein [Enterobacteria phage RB69]
Probab=93.22  E-value=2.9e-07  Score=55.85  Aligned_cols=343  Identities=10%  Similarity=0.043  Sum_probs=180.4

Q AE093565.1       21 ---------------------GVTRNAAEMFQWSKKAGVKFDIYSYDERMYNRRDAHEMDFISF------------TREN   67 (469)
Q Consensus         2 MkI~~~t~~~~p------~~GG~~~~v~~l~~~l~~~GheV~vi~~~~~~~~~~~~~~~~~~~~~~~~~~~~~~~~~~~~~~   74 (452)
                      |||+++-..+-|      ..||.|..-.+..+.|.+.||+++-++..+....+....+    +..+....+.  -......
T Consensus         1 MkIlyiPcR~VpFn~dri~GGLEaVqlN~ik~l~S~~~~IdYv~FgdenFG~~kvn~----id~pi~~KfT~~h~~ki~~   76 (366)
T gi|32350306|gb    1 MKVMFIPSRAVPFNPDRVQGGLEAVHLNVLKYLVSIGADIDYIGFDNDTFGDWKVNH----HPVGHLTKFSLGMSYTMAR   76 (366)
Confidence            999999766666      35999999999999999999999999988876665541    1112211111  1112334
                                T4 Gly-15                        T4 Arg-46 interacts with base
                                interacts beta phosphate

Q AE093565.1       68 INSTVEKLNQYDIVMFNSYPSNKFEKQAIIDFYEkvIKGVTTIKVGFMHELNKTNIdkIPYLVGIMNEMDVIYNFGEETW  147 (469)
Q Consensus        75 ~~~~~~~~~~~~DiIh~~~~~~~~~~~~~~~~~~~~~~..ip~V~t~h~~~~~~~~~~~~~..~~~~~~~~~~~~~~~~d~ii~  150 (452)
                      .+..+..-.+||.|..-.+.-+.+-++..+-.    .|.+-.+......  ...+.  ... . ..-.-.+.+.--.-++
T Consensus        77 ki~el~~~~~YDavVt~Eg~K~~~n~l~~~Gl--..~~kvrN~mATPld--P~vRg..iVq-i-W~~ai~iHknGGKnlv  146 (366)
T gi|32350306|gb   77 KIVELAGIHEYDFVVTMEPTKLTVQAIKDAGL--..SKVHKNFMATPFE--PVSRG..IVQ-I-WDQTIQIHKNGGKSYA  146 (366)
Confidence            44445566799999887776654432222111    1111111111000  00000  000 1 1112334556666778
                                                                  T4 His114, His116 and His140
                                                                  interact with glucose

Q AE093565.1      148 FSQTISDLLPSKEI--GKRTKKFTMWFNFEDLE------NNYRNKYSLD------DKSKKLVYCSRW-TTMKGPRRVLDL  212 (469)
Q Consensus       151 ~S~~~~~~~l~~~~~~~~~~~~ki~vIpnGvD~~~F~~~~~~-----~~~~r~~~~~~~~~---------~~~~~iIl~vGRl-~~~KGid~LI~A  221 (452)
                      +++..++.-.+.+.--+.++    --||++.+..-.-  +++....-+.      +..-.|+-+-|. ....--+..++|
T Consensus       147 PT~tFkdl~~~vyskmn~~~-----~vIDfDYWa~ndiis~~f~p~~li~~kp~vl~s~g~ivqAQRyD~kfRktnVAlea  222 (366)
T gi|32350306|gb  147 PTKAFREFERKYCYMTSGLT----DKIDYDYWRANPLFEAEDYPVICLNEKPEVLPATDLIISAQRYDTKMRRTDVALEA  222 (366)
Confidence            8888888777776233344    5567766653222  1222222222      233344444453 222334566777
                                                                       T4 Arg204 and Lys209
                                                                   interact with beta phosphate
```

```
Q AE093565.1      213 APMLAVKDPEFKAELKGiersigakfdifDHPNTLDCTGRTPDPNSTGTVPVYGPYIREEGIDFMAKNLFIASFYRmpka  292 (469)
Q Consensus       222 ~~~l~kk~pd~~LvIvG............~G~~~~~l~~~~~~l~l~~~V~F~G~v~~eel~~~y~~aDifV~PS~....  285 (452)
                      +++.-.+    .+-..-            +-|.-..  +.        -|+.  -.|+.++++.+..|-+.|-...
T Consensus       223 m~~~~~~----~~af~P............skWAP~k--K~--------~v~i--D~~r~~Iq~~l~~A~vlvNTCh....  270 (366)
T gi|32350306|gb  223 IKALGEN----GAGYCP............SKWAPPA--KY--------PVII--DAPHSEIMERLKTAKALINTCP....  270 (366)
Confidence              7666433    222222            2221110  00        1222  3478899999999999999998
                                                          T4 Tyr275 and Asn277,
                                                          interact with ribose
                                                           Tyr275 also interacts with base

Q AE093565.1      293 iqdYGDRMEYSQIESIAVGSIPVFDTNWGEN-NRTLDGQRyidvpysAIYSDEKD---LEGTVEKLIE-VANNKELQEKY  367 (469)
Q Consensus       286 ...~~E~~g~v~lEAMA~G~PVVat~~gG~~-evI~dG~~.......G~Lv~~~d---~~~~la~~i~~-Ll~d~~Lr~~m  350 (452)
                         ..-+-...-+||+.-|.||+-----|-+ -..++.-+       -..|+-++   ..++..+-.+ |.+=.+-.++=
T Consensus       271 ...dTGTvE~gS~EAI~kGVPVlQLiqkgy~HAT~E~DP~.......T~~V~~e~g~~~~~~ia~Y~KAl~eFtDT~E~R  340 (366)
T gi|32350306|gb  271 ...DTGTVENSSIEAISKGVPVIQLVFKDYPHATFEYDPD.......TVRVEIDSSTPKKEVVALYIKAVLEFTDTYEAR  340 (366)
Confidence                 7777788999999999999854433322 22333333       33333222   2222222222 22222333333
                            T4 Tyr307 Glu-311-interacts with ribose and conserved in all GT-B glycosyltransferases
                            and Thr308-both interact with alpha phosphate


Q AE093565.1      368 RETSYKIAKQEFDANIVLPKMFNEI  392 (469)
Q Consensus       351 g~~ar~~~~~~f~w~~va~~~~~~y  375 (452)
                      -++|-..- ++|+=+.+...|..+|
T Consensus       341 v~RA~~ly-~ky~~~~~~~mW~~~~  364 (366)
T gi|32350306|gb  341 VKRAEAVW-KKYNRDAVVAMWDKIF  364 (366)
Confidence              33333333 5666666665555544
```

Supplementary Figure S3. Fasta sequence of RB69 ORF53_52c

```
>RB69_ORF53_52c
MKKAVILGAGLATRLYPITHHIPKVLVNYKQDTILSNLYTIYSDLGADEIIVVVHSKFAE
TVRAYCEQEGFNVTIRTVDEAYGSAYALAKLYKDLDGHNVIVNWCDIIPDFGSWSWNVNA
IYVKGDECRYNFDGENITNVGSTGGNVVGIYQFKDWEFYMGSTDEEIHEYCKGRDFVEFL
YGSAFNKSELMNLIDLGDMPKLEKAHEVRELNRSFNAVEIGEETVTKFALTEHGRALQKD
EISWYLKVKSDSVPQLVSVNENNFEMERIKGKPAFEYIKSKSSLARPQIVDAILDALKFS
TDTYFVSPETVRRDFTKEFYTKVIDRCESIQPLIDSFGKITHVNYTKIGRLKPMLKQALE
HLIRYHNRSQGQYSVIHGDPNFSNTMITDNGEVKFIDPRGYFGETKIYGPKLYDEAKVLY
AVSGYDEFNANPTWGQFTIDETTCNVTININPLVYKYGKMSSFNEYHHLAVAIIWIALGG
YFKNNPLKAVAAYYKGMELLTKQLRNMGRVLQDGSISYDVAEPVTATLITKNPGKWVLTD
KETGVSYRPIGGDITHQWERI
```

Supplementary Figure S4. HHpred alignment of T4 a-gt (1XV5_A) with RB69 ORF003c (NP_861693.1).

13. **1XV5_A DNA alpha-glucosyltransferase (E.C.2.4.1.26); Transferase; HET: CME, EDO, GOL, UDP; 1.73A {Enterobacteria phage T4}; Related PDB entries: 1Y6F_A 1Y6F_B 1Y6G_A 1Y6G_B 1YA6_A 1YA6_B 1Y8Z_A 1Y8Z_B**

```
Probability: 99.9   E-value: 1.9E-24   Score: 193.53   Aligned Cols: 323   Identities: 9%   Similarity:
0.016

Q ss_pred             CeeeecCCCCCCCCccccccchHHHHHHHHHHHHHCCCEEEEEEecCCCccccccCeEEcCchhhcccCC
Q NP_861693.1 1    MKVMFIPSRAVPFNPDRVQGGLEAVHLNVLKYLVSIGADIDYIGFDNDTFGDWKVNHHPVGHLTKFSLGM   70 (366)
Q Consensus   1    mkIl~i~~~~~p~~~~~~~gG~~~~~~~l~~~L~~~g~~v~v~~~~~~~~~~~~~~~~~~~~~~~~~~~~   70 (366)
                   |||+++++.+.+      .||.++++.+++++|.++||+|++++..........................
T Consensus   2    mkIl~i~~~~~~------~gG~~~~~~~l~~~L~~~g~~v~v~~~~~~~~~~~~~~~~~~~~~~~~~~~~   65 (401)
T 1XV5_A      2    MRICIFMARGLE------GCGVTKFSLEQRDWFIKNGHEVTLVYAKDKSFTRTSSHDHKSFSIPVILAKE   65 (401)
T ss_dssp          CEEEEEETTCCC------SSHHHHHHHHHHHHHHHTTCEEEEEEECSSCCTTTTSSSCTTTCEEECTTTC
T ss_pred          CeEEEEEccCCC------CCCHHHHHHHHHHHHHHCCCEEEEEEcCCCCccccccCCcceeeccchhHHHH


Q ss_pred             chhhHHHHHHHhhhcCCCEEEEeCCCHHHHHHHHHc-----------CCCeEEEEccCCCCCCchhhHHH
Q NP_861693.1 71   SYTMARKIVELAGIHEYDFVVTMEPTKLTVQAIKDA-----------GLSKVHKNFMATPFEPVSRGIVQ   129 (366)
Q Consensus   71   ~~~~~~~~~~~~~~~~~Div~~~~~~~~~~~~~~~~-----------~~~~~~~~~~~~~~~~~~~~~~~   129 (366)
                   .....+.+      .++|+|+++.........+...           +.+.....+..............
T Consensus   66   ~~~~~~~~------~~~Dvv~~~~~~~~~~~~~~~~~~~~~~~~~~~~~~~v~~~h~~~~~~~~~~~~~~   129 (401)
T 1XV5_A      66   YDKALKLV------NDCDILIINSVPATSVQEATINNYKKLLDNIKPSIRVVVYQHDHSVLSLRRNLGLE   129 (401)
T ss_dssp          HHHHHHHH------TSCSEEEEEECCBTTSCHHHHHHHHHHHHHSCTTSEEEEEECCCSHHHHTTBSSHH
T ss_pred          HHHHHHHh------CCCCEEEEeCCCCcchhHHHHHHHHHHHHhhCCCCeEEEEEcCCcchhhhhhhcHH
```

```
Q ss_pred         HHHHhhhHHHcCCCcccccHH---HHHHHHHHhc-------cccc---ccceeccchhhcCCCCCcccCC
Q NP_861693.1 130 IWDQTIQIHKNGGKSYAPTKA---FREFERKYCY-------MTSG---LTDKIDYDYWRANPLFEAEDYP  186 (366)
Q Consensus   130 ~~~~~~~~~~~~~~~~~~s~~---~~~~~~~~~~-------~~~~---~~~~~~~~~~~~~~~~~~~~~~~~  186 (366)
                  .....      .+.+++.|..   .++....+..       ....   ++++++...+.
T Consensus   130 ~~~~~------~d~ii~~s~~~~~~~~~~~~~~~~~~~~~~~~~~~~i~~~~~~~~~~~-----------  182 (401)
T 1XV5_A      130 ETVRR------ADVIFSHSDNGDFNKVLMKEWYPETVSLFDDIEEAPTVYNFQPPMDIV-----------  182 (401)
T ss_dssp          HHHHH------CSEEEESCTTSHCCCCCHHHHSCSSCCSSSCCCCCCCEEECCCCBCHH-----------
T ss_pred          HHHHh------CCEEEecCCCcHHHHHHHHHhCcccccCCCCcccccccccCCCCccHH-----------


Q ss_pred         cccccCCCCCCCCCeEE---EEeeCCChhhcCHHHHHHHHHHHccC---cEEEeCCCCCCCC--------
Q NP_861693.1 187 VICLNEKPEVLPATDLI---ISAQRYDTKMRRTDVALEAIKALGEN---GAGYCPSKWAPPA--------  242 (366)
Q Consensus   187 ~~~~~~~~~~~~~~~~i---~~~g~~~~~~k~~~~li~a~~~l~~~---~l~i~G~~~~~~~--------  242 (366)
                    ..+.......+...+   +++|++.+ .||.+.++++++.+.+.   +++++|..  ...
T Consensus   183 --~~~~~~~~~~~~~~~~~~~~i~~~g~~~~-~K~~~~li~a~~~l~~~~~~~~~l~i~G~~--~~~~~~~~~~~~~  247 (401)
T 1XV5_A      183 --KVRSTYWKDVSEINMNINRWIGRTTT-WKGFYQMFDFHEKFLKPAGKSTVMEGLE--RSPAFIAIKEK  247 (401)
T ss_dssp          --HHHHHHCCCGGGCEEEEEEEECCSCG-GGCHHHHHHHCCCCCTTTTCEEEEECCC--CSHHHHHHHHT
T ss_pred          --HHHHHhCCCcccceeeeeEEEEccccc-ccCHHHHHHHHHHhhcCCCCEEEEEcCC--CCHHHHHHHHc


Q ss_pred         ----------------cCC---EEEcCch--HHHHHHHHhCCEEEEcCCCCC------CCCHHHHHHHHc
Q NP_861693.1 243 ----------------KYP---VIIDAPH--SEIMERLKTAKALINTCPDTG------TVENSSIEAISK  285 (366)
Q Consensus   243 ----------------~~~---v~~~g~~--~~~~~~~~~~adi~i~ps~~~e------~~~~~~~~Ea~a~  285 (366)
                                  ...   |.+.|..  +++..++..+|++++|+ ..+      ++|.+++|||++
T Consensus   248 ~~~~~~~~~~~~~~~~~~~~~~~~v~~~~~~~~~~~~~~~~l~~~~~~~~~~~~~~-~~~~~~~~~~~~~~~~~~~~~Ea~a~  316 (401)
T 1XV5_A      248 GIPYEYYGNREIDKMNLAPNQPAQILDCYINSEMLERMSKSGFGYQLS-KLNQKYLQRSLEYTHLELGAC  316 (401)
T ss_dssp          TCCEEEECGGGGGGCCCSSSCCEEEESCCCHHHHHHHHHTEEEEEECC-CCCGGGCSSCCCHHHHHHHHH
T ss_pred          CCChhHhcccchhhccCCCCCCeEEEeCcCCHHHHHHHHhcCeEEEcc-cccccccccccccHHHHHHHc
```

```
Q ss_pred             C-CCEEEecCCCCchhhcccCC-------CeEEEEcCCCCCchHHHHHHHHHHHHHhcCHHHHH---HHH
Q NP_861693.1 286 G-VPVIQLVFKDYPHATFEYDP-------DTVRVEIDSSTPKKEVVALYIKAVLEFTDTYEARV---KRA  344 (366)
Q Consensus   286 G-~pvI~~~~~~~~~~~~~~~~~~-------~g~~~~~~~~~~~~~~~~~~~~~~~~~~~~i~~l~~~~~~~~~---~~~  344 (366)
                  | +|||+++.++.. +++.++.       +|+++  ++.|     .++++++|.+++++++.+.   +++
T Consensus   317 G~~pvi~~~~~~~~~~-e~~~~~~~~~~~~~~~~~g~~~--~~~~-----~~~l~~~i~~l~~~~~~~~~~~~~~~~~~~  378 (401)
T 1XV5_A      317 GTIPVFWKSTGENL-KFRVDNTPLTSHDSGIIWF--DEND-----MESTFERIKELSSDRALYDREREKA  378 (401)
T ss_dssp          TSEEEEEHHHHHHS-BCTTTCCBGGGSCCSCEEE--CTTC-----HHHHHHHHHHHHTCHHHHHHHHHHH
T ss_pred          CCCCEEEeCCccch-hhcccCCCCccCCCCcEEE--cCCc-----hhHHHHHHHHHhcCHHHHHHHHHHH


Q ss_pred             HH-HHHHcCHHHHHHHHHHHHhC
Q NP_861693.1 345 EA-VWKKYNRDAVVAMWDKIFTA  366 (366)
Q Consensus   345 ~~-~~~~~~~~~s~~~~~~~~~~~~~~~~~~~~~~  366 (366)
                  ++ ..+.|+|+.+++++.+++++
T Consensus   379 ~~~~~~~~~~~~~~~~~~~~~~~~~~~~~~~~~~~~~~~~~~~~~~~  401 (401)
T 1XV5_A      379 YEFLYQHQDSSFCFKEQFDIITK  401 (401)
T ss_dssp          HHHHHHHHBHHHHHHHHHHHHTC
T ss_pred          HHHHHHcCCHHHHHHHHHHHhhC
```

Supplementary Figure S5. HHpred alignment of *Paramecium bursaria* Chlorella virus NY2A glycosyltransferase B736L (3OY2_A) with RB69 ORF003c (NP_861693.1).

3. **3OY2_A Glycosyltransferase B736L; Rossmann fold, glycosyltransferase, GDP-mannose, sugar; 2.31A {Paramecium bursaria Chlorella virus NY2A}; Related PDB entries: 3OY2_B 3OY7_A 3OY7_B**

Probability: 99.93   E-value: 4.0E-26   Score: 205.31   Aligned Cols: 330   Identities: 12%   Similarity: 0.018

```
Q ss_pred         CeeeecCCCCCCCCccccccchHHHHHHHHHHHHHCCCEEEEEEecCCCccccccCeEEcC--chhhccc
Q NP_861693.1 1   MKVMFIPSRAVPFNPDRVQGGLEAVHLNVLKYLVSIGADIDYIGFDNDTFGDWKVNHHPVG--HLTKFSL  68 (366)
Q Consensus   1   mkIl~i~~~~~p~~~~~~~gG~~~~~~~l~~~L~~~g~~v~v~~~~~~~~~~~~~~~~~~~~~--~~~~~~~  68 (366)
                  |||+++++.+.+      .||.+.++..+++.|.+ ||+|++++.................  .......
T Consensus   1   mkIl~i~~~~~~------~gG~~~~~~~l~~~L~~-g~~v~v~~~~~~~~~~~~~~~~~~~~~~~~~~~~~~  63 (413)
T 3OY2_A      1   MKLIIVGAHSSV------PSGYGRVMRAIVPRISK-AHEVIVFGIHAFGRSVHANIEEFDAQTAEHVRGL  63 (413)
T ss_dssp         CEEEEEEECTTC------CSHHHHHHHHHHHHHTT-TSEEEEEEESCCSCCSCSSSEEEEHHHHHHHTTC
T ss_pred         CeEEEEecCCCC------CCcHHHHHHHHHHHHHc-cCEEEEEEEcCCCceecceeeeecCccceecCCc


Q ss_pred         CCchhhHHHHHHHhhhcCCCEEEEeCCCHHHHHHHHHcCCCeEEEEccCCCCCCchhhHHHHHHHhhhHH
Q NP_861693.1 69  GMSYTMARKIVELAGIHEYDFVVTMEPTKLTVQAIKDAGLSKVHKNFMATPFEPVSRGIVQIWDQTIQIH  138 (366)
Q Consensus   69  ~~~~~~~~~~~~~~~~~~~~~Div~~~~~~~~~~~~~~~~~~~~~~~~~~~~~~~~~~~~~~~~~~~~~~~~~  138 (366)
                  .........+.+.++..++|+|+++.........+...........+............  .+.......
T Consensus   64  ~~~~~~~~~l~~~~~~~~~~~Div~~~~~~~~~~~~~~~~~~~~~~~~~~v~~~~~~~~~~~--~~~~~~~~~  131 (413)
T 3OY2_A      64  NEQGFYYSGLSEFIDVHKPDIVMIYNDPIVIGNYLLAMGKCSHRTKIVLYVDLVSKNIR--ENLWWIFSH  131 (413)
T ss_dssp         CSTTCCHHHHHHHHHHHCCSEEEEEECHHHHHHHHHHGGGCCSCCEEEEEECCCSBSCC--GGGGGGGGC
T ss_pred         CcCCcchhhHHHHHHHHCCCEEEEECChhHHHHHHHHhhhccCCCcEEEEEcCCccchh--hhhHHHhcC
```

```
Q ss_pred             HcCCCcccccHHHHHHHHHHhccccc--ccceecccchhhcCCCCCcccCCccccccCCCCCCC--CCeEEE
Q NP_861693.1 139 KNGGKSYAPTKAFREFERKYCYMTSG--LTDKIDYDYWRANPLFEAEDYPVICLNEKPEVLP--ATDLII  204 (366)
Q Consensus   139 ~~~~~~~~~~s~~~~~~~~~~~~~~~~~~--~~~~~~~~~~~~~~~~~~~~~~~~~~~~~~~~~~~~~~--~~~~i~  204 (366)
                  ...+.+++.|....+....+......  ++++++...+.             ..........  +...++
T Consensus   132 ~~~d~vi~~s~~~~~~~~~~~~~~~~~~~~~~ip~~~~~~~~~~~~~~~-------------~~~~~~~~~~~~~~~~~~i~  188 (413)
T 3OY2_A      132 PKVVGVMAMSKCWISDICNYGCKVPINIVSHFVDTKTIY-------------DARKLVGLSEYNDDVLFL  188 (413)
T ss_dssp           TTEEEEEEESSTHHHHHHHHTTCCSCEEECCCCCCCCCCT-------------THHHHTTCGGGTTSEEEE
T ss_pred            cCCCEEEEcCHHHHHHHHHcCCCCCeEEEeCCCCchHhh-------------hHHHHhCCCccCCCEEEE


Q ss_pred             EeeCCChhhcCHHHHHHHHHHHccC----cEEEeCCCCCCCCc---------------CC---EEEcC--
Q NP_861693.1 205 SAQRYDTKMRRTDVALEAIKALGEN----GAGYCPSKWAPPAK---------------YP---VIIDA--  250 (366)
Q Consensus   205 ~~g~~~~~~~~k~~~~~li~a~~~~l~~~~----~~l~i~G~~~~~~~~~~~~~~---------------~~~~----v~~~~g--  250 (366)
                  ++|++.+ .||++.++++++.+.+.    +++++|.+  +...                ..   |+++|
T Consensus   189 ~~g~~~~~-~k~~~~~li~~~~~~~l~~~~~~~~~~~~~~~~l~i~G~~--~~~~~~~~~~~~~~~~l~~~~~~~~~~~~~~~~v~~~~g~v  255 (413)
T 3OY2_A      189 NMNRNTA-RKRLDIYVLAAARFISKYPDAKVRFLCNS--HHESKFDLHSIALRELVASGVDNVFTHLNKI  255 (413)
T ss_dssp           CCSCSSG-GGTHHHHHHHHHHHHHHCTTCCEEEEEEC--CTTCSCCHHHHHHHHHHHHTCSCHHHHHTTE
T ss_pred            EcCCCch-hcCHHHHHHHHHHHHHHhCCCcEEEEeeCC--CCcccccHHHHHHHHHHHCCCcceEEecchh


Q ss_pred             -----ch--HHHHHHHHhCCEEEEcCCCCCCCCHHHHHHHHcCCCEEEecCCCCchhhcccCCCeEEEEc
Q NP_861693.1 251 -----PH--SEIMERLKTAKALINTCPDTGTVENSSIEAISKGVPVIQLVFKDYPHATFEYDPDTVRVEI  313 (366)
Q Consensus   251 -----~~--~~~~~~~~~~~~~~~~adi~i~ps~~~~e~~~~~~~~~~~~Ea~a~G~pvI~~~~~~~~~~~~~~~~~~~~~~~~~~~~g~~~~~~~  313 (366)
                       .+  +++.++++.||++++|+ ..|++|.+++|||++|++|||+++.++.. +++. +.+|+++
T Consensus   256 ~~~~~~~~~~~~~~~~~~~~~~~~~~~~~adi~l~~s-~~e~~~~~~~~~~Ea~~~G~pvi~~~~~~~~~~~~-~~~~-~~~~g~~~~~--  320 (413)
T 3OY2_A      256 MINRTVLTDERVDMMYNACDVIVNCS-SGEGFGLCSAEGAVLGKPLIISAVGGAD-DYFS-GDCVYKI--  320 (413)
T ss_dssp           EEECSCCCHHHHHHHHHHCSEEEECC-SCCSSCHHHHHHHTTTCCEEEECCHHHH-HHSC-TTTSEEE--
T ss_pred            cccCCCCCHHHHHHHHHhCCEEEEcC-CCCCCCHHHHHHHHhCCCEEEeCCCCch-hhcc-CCceEEe--
```

```
Q ss_pred             CC------------------CCCchHHHHHHHHHHHHHhcCHHHHH---HHHHH-HHHHcCHHHHHHHHH
Q NP_861693.1 314 DS------------------STPKKEVVALYIKAVLEFTDTYEARV---KRAEA-VWKKYNRDAVVAMWD  361 (366)
Q Consensus   314 ~~------------------~~~~~~~~~~~~~~i~~l~~~~~~~~---~~~~~-~~~~~s~~~~~~~~~  361 (366)
                      ++                  .|     .++++++|. ++.+++.+.   +++++ ..+.|+|+.+++++.
T Consensus   321 ~~~~~~~~~~~~~~~~~~~~~~-----~~~l~~~i~-~~~~~~~~~~~~~~~~~~~~~~~s~~~~~~~~~  384 (413)
T 3OY2_A      321 KPSAWISVDDRDGIGGIEGIID-----VDDLVEAFT-FFKDEKNRKEYGKRVQDFVKTKPTWDDISSDII  384 (413)
T ss_dssp            CCCEEEECTTTCSSCCEEEECC-----HHHHHHHHH-HTTSHHHHHHHHHHHHHHHTTSCCHHHHHHHHH
T ss_pred             CccceeccCCCCCCCCcccCCC-----HHHHHHHHH-HhcCHHHHHHHHHHHHHHHhhCCCHHHHHHHHH


Q ss_pred             HHHhC
Q NP_861693.1 362 KIFTA  366 (366)
Q Consensus   362 ~~~~~  366 (366)
                      +++.+
T Consensus   385 ~~~~~  389 (413)
T 3OY2_A      385 DFFNS  389 (413)
T ss_dssp            HHHHH
T ss_pred             HHHHH
```

Supplementary Figure S6. HHpred alignment of *Halothermothrix orenii* glycosyltransferase B736L (2R60_A) with RB69 ORF003c (NP_861693.1).

5. **2R60_A Glycosyl transferase, group 1 (E.C.2.4.1.14); Rossmann-fold, Transferase; 1.8A {Halothermothrix orenii}; Related PDB entries: 2R66_A 2R68_A**

Probability: 99.92   E-value: 5.6E-26   Score: 209.22   Aligned Cols: 346   Identities: 12%   Similarity: -0.014

```
Q ss_pred            CeeeecCCCC----------CCCCcccccccchHHHHHHHHHHHHHCCCEEEEEEecCCCc----------
Q NP_861693.1 1     MKVMFIPSRA----------VPFNPDRVQGGLEAVHLNVLKYLVSIGADIDYIGFDNDTF----------  50 (366)
Q Consensus   1     mkIl~i~~~~----------~p~~~~~~~gG~~~~~~~l~~~L~~~g~~v~v~~~~~~~~~----------  50 (366)
                     |||+++++.+          .+..    .||.+.++.+++++|.++||+|++++......
T Consensus   8     ~kIl~i~~~~~~~~~~~~~~~~~~----~gG~~~~~~~l~~~L~~~G~~V~v~~~~~~~~~~~~~~~~~~~~~  73 (499)
T 2R60_A      8     KHVAFLNPQGNFDPADSYWTEHPD----FGGQLVYVKEVSLALAEMGVQVDIITRRIKDENWPEFSGEID  73 (499)
T ss_dssp           CEEEEECCSSCCCTTCTTTTSBTT----BSHHHHHHHHHHHHHHHTTCEEEEEEECCCBTTBGGGCCSEE
T ss_pred            ceEEEECCCcCCCcccccccCCCC----CCcHHHHHHHHHHHHHHCCCcEEEEeCCCCCCCchhhcCCcc


Q ss_pred            ---cccccCeEEcC------chhhcccCCchhhHHHHHHHhhhcC--CCEEEEeCCCHHHHHHHHHcCCC
Q NP_861693.1 51    ---GDWKVNHHPVG------HLTKFSLGMSYTMARKIVELAGIHE--YDFVVTMEPTKLTVQAIKDAGLS  109 (366)
Q Consensus   51    ---~~~~~~~~~~~------~~~~~~~~~~~~~~~~~~~~~~~~~--~Div~~~~~~~~~~~~~~~~~~~~  109 (366)
                        .........+.      ................+.+.++..+  +|+|+++.........+......
T Consensus   74    ~~~~~~~~~~~~~~~~~~~~~~~~~~~~~~~~~~~~~~~~~~~~~~dii~~~~~~~~~~~~~~~~~~~~  143 (499)
T 2R60_A      74    YYQETNKVRIVRIPFGGDKFLPKEELWPYLHEYVNKIINFYREEGKFPQVVTTHYGDGGLAGVLLKNIKG  143 (499)
T ss_dssp           ECTTCSSEEEEEECCSCSSCCCGGGCGGGHHHHHHHHHHHHHHHTCCCSEEEEEHHHHHHHHHHHHHHHC
T ss_pred            cceeeCCeEEEEcCCCCCcCCChHHhHHHHHHHHHHHHHHHHHHCCCCcEEEEcCccccHHHHHHHHhhC
```

```
Q ss_pred         eEEEEccCCCCCCch----------------hhHHHHHHHhhhHHHcCCCcccccHHHH------HHHHH
Q NP_861693.1 110 KVHKNFMATPFEPVS----------------RGIVQIWDQTIQIHKNGGKSYAPTKAFR------EFERK  157 (366)
Q Consensus   110 ~~~~~~~~~~~~~~~----------------~~~~~~~~~~~~~~~~~~~~~~~s~~~~------~~~~~  157 (366)
                  ..+............                ..............+..+.+++.|....      .....
T Consensus   144 ~~~i~~~h~~~~~~~~~~~~~~~~~~~~~~~~~~~~~~~~~~~~~~~~d~ii~~s~~~~~~~~~~~~~~~~  213 (499)
T 2R60_A      144 LPFTFTGHSLGAQKMEKLNVNTSNFKEMDERFKFHRRIIAERLTMSYADKIIVSTSQERFGQYSHDLYRG  213 (499)
T ss_dssp         CCEEEECSSCHHHHHHTTCCCSTTSHHHHHHHCHHHHHHHHHHHHHHCSEEEESSHHHHHHTTTSGGGTT
T ss_pred         CCEEEEecCcchhhhhHhcCCcccchhhhHHHHHHHHHHHHHHHHHhCCEEEEeCchhHhhhchhhhhcC


Q ss_pred         -Hhc-cccc---ccceeccchhhcCCCCCcccCCcccccCCCCCC-CCCeEEEEeeCCChhhcCHHHHHH
Q NP_861693.1 158 -YCY-MTSG---LTDKIDYDYWRANPLFEAEDYPVICLNEKPEVL-PATDLIISAQRYDTKMRRTDVALE  221 (366)
Q Consensus   158 -~~~-~~~~---~~~~~~~~~~~~~~~~~~~~~~~~~~~~~~~~~-~~~~~i~~~g~~~~~~k~~~~li~  221 (366)
                   +.. ....   ++++++...+................+...+.. .+...++|+|++.+ .||++.+++
T Consensus   214 ~~~~~~~~~~~~i~~~~~~~~~~~~~~~~~~~~~~~~~~~~~~~~~~~~~~i~~~G~~~~-~k~~~~li~  282 (499)
T 2R60_A      214 AVNVEDDDKFSVIPPGVNTRVFDGEYGDKIKAKITKYLERDLGSERMELPAIIASSRLDQ-KKNHYGLVE  282 (499)
T ss_dssp         TCCTTCGGGEEECCCCBCTTTSSSCCCHHHHHHHHHHHHHHSCGGGTTSCEEEECSCCCG-GGCHHHHHH
T ss_pred         CCCCCCCCCEEEECCCCCHhhcCCccchHHHHHHHHHHHHhhCCCCCCCCEEEEEecCCc-ccCHHHHHH


Q ss_pred         HHHHHccC----cEEEeCCC--------------------------------CCCCCcCCEEEcCch--H
Q NP_861693.1 222 AIKALGEN----GAGYCPSK--------------------------------WAPPAKYPVIIDAPH--S  253 (366)
Q Consensus   222 a~~~l~~~----~l~i~G~~--------------------------------~~~~~~~~v~~~g~~--~  253 (366)
                  ++..+.+.    ++.++|.+                                ..      |.+.|.+  +
T Consensus   283 a~~~l~~~~~~~~~~~~i~g~g~~~~~~~~~~~~~~~~~~~~~~~~~~~~~~~~~~------v~~~g~~~~~  346 (499)
T 2R60_A      283 AYVQNKELQDKANLVLTLRGIENPFEDYSRAGQEEKEILGKIIELIDNNDCRGK------VSMFPLNSQQ  346 (499)
T ss_dssp         HHHTCHHHHHHCEEEEEESSCSBTTTBCTTSCHHHHHHHHHHHHHHHHTTCBTT------EEEEECCSHH
T ss_pred         HHHHHHHhcccccEEEEEecCCCcchhhhhcChhHHHHHHHHHHHHhcCCCCCc------EEEccCCCHH
```

```
Q ss_pred              HHHHHHH----hCCEEEEcCCCCCCCCHHHHHHHHcCCCEEEecCCCCchhhcccCCCeEEEEcCCCCCc
Q NP_861693.1 254 EIMERLK----TAKALINTCPDTGTVENSSIEAISKGVPVIQLVFKDYPHATFEYDPDTVRVEIDSSTPK  319 (366)
Q Consensus   254 ~~~~~~~----~adi~i~ps~~~e~~~~~~~~Ea~a~G~pvI~~~~~~~~~~~~~~~~~~~~~g~~~~~~~~~~~~  319 (366)
                  ++.++++    .||++++|+ ..|++|.+++|||++|+|||+++.++.. +++.++.+|+++  ++.|
T Consensus   347 ~~~~~~~~~~~~~~di~i~ps-~~e~~~~~~~~Ea~a~G~PvI~~~~~~~~~~-e~i~~~~~g~~~--~~~~--  410 (499)
T 2R60_A      347 ELAGCYAYLASKGSVFALTS-FYEPFGLAPVEAMASGLPAVVTRNGGPA-EILDGGKYGVLV--DPED--  410 (499)
T ss_dssp         HHHHHHHHHHHTTCEEEECC-SCBCCCSHHHHHHHTTCCEEEESSBHHH-HHTGGGTSSEEE--CTTC--
T ss_pred         HHHHHHHHHHhcCcEEEEcC-CCCCCChHHHHHHHcCCCEEEeCCCChH-HhccCCCCEEEe--CCCC--

Q ss_pred         hHHHHHHHHHHHHHhcCHHHHH---HHHHH-HHHHcCHHHHHHHHHHHHHhC
Q NP_861693.1 320 KEVVALYIKAVLEFTDTYEARV---KRAEA-VWKKYNRDAVVAMWDKIFTA  366 (366)
Q Consensus   320 ~~~~~~~~~~~~i~~l~~~~~~~~~~~---~~~~~-~~~~~s~~~~~~~~~~~~~~~~~~  366 (366)
                     .++++++|.+++++++.+.   +++++ ..+.|+|+.+++++.+++++
T Consensus   411 ---~~~l~~~i~~l~~~~~~~~~~~~~~~~~~~~~~~~~~~~s~~~~~~~~~~~~~~~~~~  458 (499)
T 2R60_A      411 ---PEDIARGLLKAFESEETWSAYQEKGKQRVEERYTWQETARGYLEVIQE  458 (499)
T ss_dssp         ---HHHHHHHHHHHHSCHHHHHHHHHHHHHHHHHHSBHHHHHHHHHHHHHH
T ss_pred         ---HHHHHHHHHHHhCCHHHHHHHHHHHHHHHHHhCCHHHHHHHHHHHHHH
```

Supplementary Figure S7. SDS-PAGE of *E. coli* DH10B containing in Lane (1) pHERD20T plasmid with the region of the RB69 genome encompassing ORF53c and ORF52c and (2) pHERD20T plasmid alone. Both cultures were induced with 1% arabinose for 1 hour at 37°C. Black arrow in indicates a protein band with a mass consistent to the predicted mass of the full length RB69 ORF53_2c (63.9 kDa).

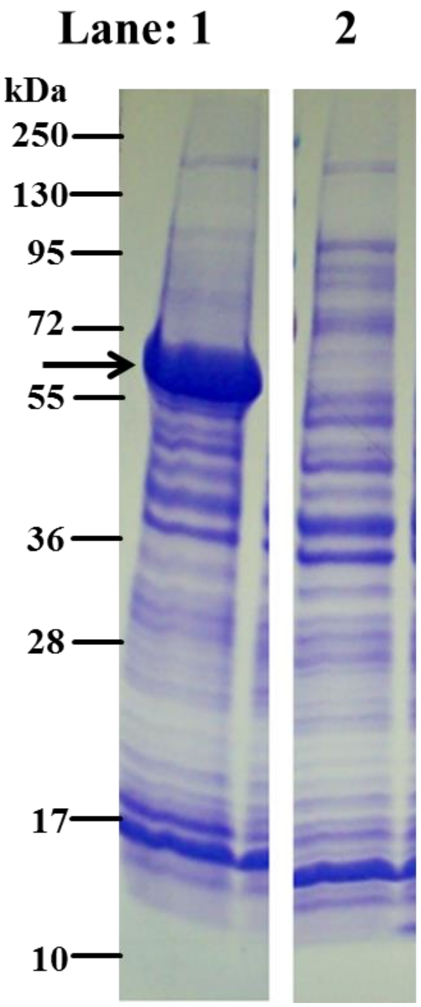

Supplement: Supplementary file 1 [file viruses-10-00313-s001.pdf]
